# Salusin-β mediates tubular cell apoptosis in acute kidney injury: Involvement of the PKC/ROS signaling pathway

**DOI:** 10.1016/j.redox.2019.101411

**Published:** 2019-12-20

**Authors:** Qing-Bo Lu, Qiong Du, Hui-Ping Wang, Zi-Han Tang, Yuan-Ben Wang, Hai-Jian Sun

**Affiliations:** a Department of Neurology, Affiliated ZhongDa Hospital, School of Medicine, Southeast University, Nanjing, Jiangsu, 210009, PR China; b Department of Basic Medicine, Wuxi School of Medicine, Jiangnan University, Wuxi, Jiangsu, 214122, PR China; c Yong Loo Lin School of Medicine, National University of Singapore, Singapore, 117597, Singapore

**Keywords:** Apoptosis, Oxidative stress, DNA damage, Salusin, LPS, Cisplatin

## Abstract

Salusin-β is abundantly expressed in many organs and tissues including heart, blood vessels, brain and kidneys. Recent studies have identified salusin-β as a bioactive peptide that contributes to various diseases, such as atherosclerosis, hypertension, diabetes and metabolic syndrome. However, the role of salusin-β in the pathogenesis of acute kidney injury (AKI) is largely unclear. In the present study, we investigated the roles of salusin-β in cisplatin or lipopolysaccharide (LPS)-induced renal injury. Herein, we found that salusin-β expression was upregulated in both renal tubular cells and kidney tissues induced by both cisplatin and LPS. *In vitro*, silencing of salusin-β diminished, whereas overexpression of salusin-β exaggerated the increased PKC phosphorylation, oxidative stress, histone γH2AX expression, p53 activation and apoptosis in either cisplatin or LPS-challenged renal tubular cells. More importantly, salusin-β overexpression-induced tubular cell apoptosis were abolished by using the PKC inhibitor Go 6976, reactive oxygen species (ROS) scavenger NAC, nicotinamide adenine dinucleotide phosphate (NADPH) oxidase inhibitor apocynin (Apo) or p53 inhibitor Pifithrin-α. In animals, blockade of salusin-β alleviated PKC phosphorylation, ROS accumulation, DNA damage, and p53 activation as well as renal dysfunction in mice after administration of cisplatin or LPS. Taken together, these results suggest that overexpressed salusin-β is deleterious in AKI by activation of the PKC/ROS signaling pathway, thereby priming renal tubular cells for apoptosis and death.

## Highlights

• Both cisplatin and LPS elevated the renal levels of endogenous salusin-β in mice.
• Treatment with neutralizing the salusin-β antibody ameliorated renal dysfunction and tubular cell DNA damage and apoptosis.
• Deletion of salusin-β alleviated, while overexpression of salusin-β aggravated tubular epithelial cell DNA damage and apoptosis.
• Salusin-β contributed to tubular cell DNA damage and apoptosis through the PKC/ROS signaling pathway.

## Graphical abstract

**Figure:**
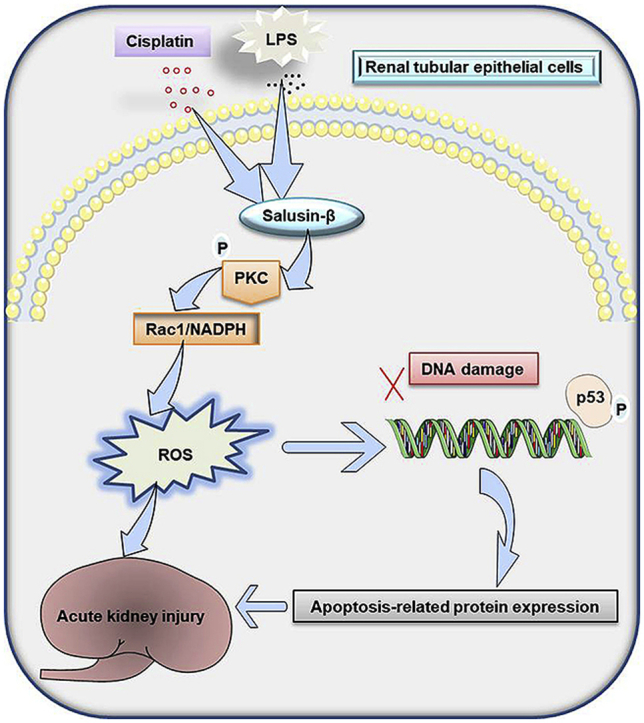

## 1 Introduction

Acute kidney injury (AKI) is manifested by a sharp decline in renal function that threats millions of patients with high mortality and morbidity [1]. Currently, it is believed that several factors are involved in the pathogenesis of AKI, including renal venous congestion, inflammatory response, oxidative stress, coagulation cascade activation, renal hypoperfusion and microcirculatory disturbance [[2], [3], [4]]. Despite of the current understanding of the pathophysiological mechanisms of AKI is constantly growing; the effective pharmacological strategies for the treatment of AKI are still not available [5].

Acute tubular epithelial cell apoptosis is an important characteristic of AKI induced by various stimuli, such as hypoxic environment, chemical drugs, hemodynamic changes and mechanical stress [5,6]. Cisplatin is one of the most widely used chemotherapeutic drugs to treat solid tumors in multiple organs such as lung, bladder, ovarian, head and neck, cervical, ovaries, and testicular [7]. However, severe adverse effects are observed in normal tissues, notably nephrotoxicity in the kidneys [8]. It is reported that more than 30% of patients may suffer from the symptoms of AKI after administration of cisplatin [9]. The underlying mechanisms of cisplatin-induced AKI are very complicated, but massive renal proximal tubular cell death, including cell necrosis and apoptosis, nuclear DNA injury, oxidative stress inflammatory response and activation of apoptotic cascades are involved [10,11]. Epidemiological analysis has shown that sepsis is a major cause for AKI in patients, and the mortality of sepsis-related AKI is up to 70% [3,12]. As an important component of cell wall of most Gramnegative bacteria, lipopolysaccharide (LPS) is able to mimic sepsis-related AKI in animals via triggering enormous cytokine synthesis, excessive oxidative stress, renal hypoperfusion, which eventually leads to a rapid decline in renal function [13]. As a result, in this study, both cisplatin and LPS were used to establish two rodent models of AKI.

Salusin-β, a multifunctional bioactive peptide with 20 amino acid residues [14], plays a critical role in atherosclerosis, hypertension, and metabolic syndrome [15]. Salusin-β is widely expressed in the brain, heart, blood vessels, livers and kidneys [[16], [17], [18]]. The circulating levels of salusin-β are particularly higher in patients with coronary artery disease, cerebrovascular disease, diabetes mellitus, and hypertension than that of control healthy subjects [19,20]. From cellular, animal, and clinical experiments, salusin-β exerts a pro-atherogenic role via promoting macrophage foam cell formation [21]. Salusin-β is a potential stimulator for induction of oxidative stress and inflammatory response in cardiovascular system [22,23]. Overexpression of salusin-β contributes the development of hypertension [24] and atherosclerosis [25]. By contrast, silencing of salusin-β ameliorated the progress of heart failure [26], hypertension [27], and intimal hyperplasia after vascular injury [28]. Likewise, blockade of salusin-β with its specific antibody improves pulmonary arterial hypertension [29], attenuates hypertension and cardiac hypertrophy [30], and enhances angiogenesis after myocardial ischemia reperfusion injury [31]. These existing studies have shown that endogenous salusin-β may be a critical regulator under disease conditions. However, whether treatment with salusin-β antibody could ameliorate AKI is still unknown. Given that salusin-β is a fundamental contributor to oxidative stress and inflammatory response, and both of which are critically involved in the pathogenesis of AKI. Thus, we hypothesized that salusin-β might participate in the process of AKI in association with elevated oxidative stress and inflammatory response. Therefore, in the present study, we investigated whether salusin-β is induced in the kidneys from cisplatin or LPS-treated mice. By using gain- and loss-of-function approaches, we also sought to examine the potential roles of salusin-β in cisplatin or LPS-induced nephropathy, as well as the underlying mechanisms.

## 2 Materials and methods

### 2.1 Reagents and antibodies

Dulbecco's modified Eagle's medium (DMEM), penicillin and streptomycin antibiotic mixture, and fetal bovine serum (FBS) were obtained from Gibco BRL (Carlsbad, CA, USA). N-acetyl-cysteine (NAC), dhydroethidium (DHE), EHT1864, cisplatin, NADPH inhibitor apocynin, Pifithrin-α, LPS, 2,7-dichlorofluorescein diacetate (DCFH-DA), 4′,6-diamidino-2-phenylindole (DAPI) were purchased from Sigma-Aldrich (St Louis, MO. USA). Adenoviral constructs carrying shRNA against salusin-β and a control shRNA (a negative control) were constructed by Genomeditech Co. (Shanghai, China) according to our previous reports [22,28]. Recombinant lentivirus vector expressing salusin-β was designed and identified by Invitrogen (Life Tech, Shanghai, China) as we previously described [23,24]. Antibodies against NOX4 (67 kDa), p22^phox^ (22 kDa), p47^phox^ (47 kDa), nitrotyrosine (50 kDa), β-actin (42 kDa), α 1 Sodium Potassium ATPase (NKAα1, 112 kDa), PKC (75 kDa), phosphorylated PKC (T514, 75 kDa), F4/80 (a macrophage marker), horseradish peroxidase (HRP)-conjugated secondary antibodies and a PKC inhibitor Go 6976 were purchased from Abcam (Cambridge, MA, USA). Antibodies against cleaved-caspase-3 (17 kDa), cleaved PARP (89 kDa), Bax (20 kDa), Bcl-2 (26 kDa), γH2AX (15 kDa), H2AX (15 kDa), phosphorylated and total p53 (53 kDa) were obtained by Cell Signaling Technology (Danvers, MA, USA). Antibody against salusin-β (36 kDa) was obtained Wuhan USCN Business Co., Ltd. (Wuhan, China). Antibody against megalin was purchased from Santa Cruz Biotechnology (Santa Cruz, CA. USA). Click-iT™ Plus TUNEL Assay for In Situ Apoptosis Detection, Alexa Fluor™ 488 dye and Alexa Fluor™ 594 dye were purchased from Invitrogen (Carlsbad, CA, USA). The specific primers and siRNA sequences were synthesized by Sangon Biotech Co., Ltd (Shanghai, China). Immunohistochemistry kit and diaminobenzidine (DAB) were obtained from Boster Biological Technology Co., Ltd (Wuhan, China). Annexin V-FITC/PI Apoptosis Detection Kit was purchased from Shanghai Yisheng technology (Shanghai, China). The doses of chemicals were selected according to our preliminary results and previous reports [28,[32], [33], [34], [35]].

### 2.2 Animal models

Wild-type 8-week-old male C57BL/6 mice were purchased from Model Animal Research Center of Nanjing University (Nanjing, China). All animal experiments were in accordance with to the regulations of the Experimental Animal Care and Use Committee of Jiangnan University. All procedures were complied with the Guide for the Care and Use of Laboratory Animal published by the National Institutes of Health (NIH publication, 8th edition, 2011). All mice were housed in a temperature controlled and humidity-controlled room under a 12 h: 12 h light/dark cycle, and the mice were allowed free access to water and standard chow *ad libitum*. In cisplatin-induced acute kidney injury, the mice were subjected to cisplatin (Sigma-Aldrich, St. Louis, MO, USA) by a single intraperitoneal (i.p.) injection (20 mg/kg body weight) [33,36]. The LPS-induced AKI model was produced by a single intraperitoneal (i.p.) injection with 20 mg/kg body weight of LPS to induce septic AKI according to previous reports [37,38]. To determine the protective effect of salusin-β antibody in AKI models, the mice were pretreated with the control antibody or neutralizing salusin-β antibody for 48 h at a dose of 0.1 mg/kg/day via i.p. injection before cisplatin (5 days duration) or LPS (3 days duration) treatment. The dose of salusin-β antibody used in the present study was selected according to the previous reports [29,31]. In cisplatin-induced acute kidney injury mice, the mice were sacrificed after cisplatin administration for 72 h. In LPS-induced nephropathy, the mice were sacrificed after LPS administration for 24 h. The serum or plasma was collected and stored at −80 °C for further analysis. The kidney tissues were fixed in 4% paraformaldehyde for histology analysis. The remaining kidney tissue was stored at −80 °C for biochemical analysis.

### 2.3 Histology and immunohistochemistry

For histology analysis, paraffin kidney sections were cut at 5 μm and stained with HE (haematoxylin and eosin) and periodic acid-Schiff staining (PAS) staining. The images were captured using a light microscope (Zeiss, Jena, Germany). The degree of tubular damage were graded by a semi-quantitative score in terms of tubular dilation, cast formation, tubular atrophy, brush border loss, and the percentage of tubules in the external medulla area where epithelial necrosis, according to previous studies [39].

For immunohistochemistry, after incubation with 5% normal goat serum, sections were incubated with primary antibodies F4/80 in humidified chambers overnight at 4 °C. Then the sections were incubated with horseradish peroxidase-coupled secondary antibody for 1 h at room temperature. Sections were incubated with 3,3′-diaminobenzidine to give a brown reaction product. Sections were then counterstained with haematoxylin, dehydrated and covered, and the images were captured using a light microscope (Zeiss, Jena, Germany).

### 2.4 Renal function assessment

To evaluate kidney function, we measured serum blood urea nitrogen (BUN) and creatinine (Cre) with commercial kits (Nanjing Jiancheng Bioengineering Institute, Nanjing, China) according to the manufacturer's protocols. The commercial kit for measurement of Cre was based on creatinine acid oxidase method as previous reports [40,41]. The levels of urine Fg were measured using commercially available species-specific Luminex-based assay kit from Millipore (Billerica, MA, USA). The levels of serum Cystatin C were analyzed by a mouse Cystatin C commercial kit (Elabscience Biotechnology Co.,Ltd, Wuhan, China). Subsequently, the optical density for measurement of serum Cystatin C was measured using a microplate reader (SYNERGY H4, BioTek, VT, USA) at the absorbance of 450 nm.

### 2.5 Cell culture

A human proximal tubule epithelial cell line (HK-2) was purchased from the American Type Culture Collection (ATCC, Manassas, VA, USA). The cells were cultured in DMEM/F-12 medium that was supplemented with 10% FBS and antibiotics (100 units/ml penicillin and 100 mg/ml streptomycin) in a humidified atmosphere of 5% CO_2_ at 37 °C. For determination of cisplatin or LPS on the expression of salusin-β at different time points, the cells were incubated with cisplatin (0, 5, 10, 20, 40 μM) or LPS (0, 1 3, 10, 30 μg/ml) for 6, 12, 24, 48 h, respectively. For silencing of salusin-β *in vitro*, HK-2 cells were transfected with adenovirus mediated shRNA against salusin-β or control shRNA (MOI = 100) for 48 h, and then used for cisplatin or LPS stimulation. For overexpression of salusin-β *in vitro*, HK-2 cells were transfected with lentivirus expressing salusin-β or lentiviral vector (MOI = 100) and grown in 5% CO_2_ incubator at 37 °C for 48 h before administration of cisplatin or LPS. Cells were harvested at the required time points.

### 2.6 Cell viability, apoptosis and LDH release

The cell viability was measured by using Cell Counting Kit-8 (CCK-8, Dojindo, Kumamoto, Japan) in accordance with the manufacturer's protocols. In brief, after being treated, 10 μL of CCK-8 solution were added to the required wells for 2 h at 37 °C. Subsequently, the optical density was measured using a microplate reader (SYNERGY H4, BioTek, VT, USA) at the absorbance of 450 nm. Lactate dehydrogenase (LDH) assay was carried out with the aid of a LDH-cytotoxicity assay kit (Beyotime Institute of Biotechnology, Shanghai, China) following the manufacturer's instructions, and the optical density was measured using a microplate reader (SYNERGY H4, BioTek, VT, USA) at the absorbance of 450 nm. For assessment of cell apoptosis, a commercial kit of Click-iT® TUNEL Alexa Fluor® 594 Imaging Assay (Thermo Fisher Scientific Inc, Waltham, MA. USA) was used. According to the manufacturer's protocols, the red or blue fluorescence was measured with Nikon Eclipse 80i fluorescence microscope (Japan). Furthermore, cell apoptosis was also assessed by a flow cytometry (Accuri C6, BD Biosciences) by using Annexin V-FITC/PI apoptosis detection kit as we previously described [42].

### 2.7 Caspse-3 activity

The caspase-3 activity was tested by using the caspase-3 activity assay kit (Beyotime Institute of Biotechnology, Shanghai, China) according to the manufacturer's instructions. In short, the protein level in collected samples was determined by using BCA colorimetric protein kit (Beyotime Biotechnology, Shanghai, China). For each sample, equal amount of protein (200 μg) was mixed with reaction buffer (50 μL) and caspase-3 substrate (5 μL) in the darkness at 37 °C for 4 h. The absorbance at 405 nm was measured by using a microplate reader (SYNERGY H4, BioTek, VT, USA).

### 2.8 Measurement of ROS generation *in vivo* and *in vitro*

The intracellular ROS in HK-2 cells or renal tissues were determined with two fluorescent probes, DHE and DCFH-DA, respectively. The collected samples were incubated with DHE (10 μM) or DCFH-DA (10 μM) for 30 min in a light-protected humidified chamber. The fluorescence signals were captured and quantified with the Image-Pro Plus software (Version 6.0, Media Cybernetics, Bethesda, MD, USA) by using the same parameters.

### 2.9 Measurement of oxidative stress markers

The levels of malondialdehyde (MDA, A003-1-2, thiobarbituric acid method) and activities of superoxide dismutase (SOD, A001-1-2, hydroxylamine method), catalase (CAT, A007-1-1, visible light method) and glutathione peroxidase (GSH, A005-1-2, colorimetric method) were determined using commercial assay kits following the manufacturer's instructions (Nanjing Jiancheng Bioengineering Institute, Nanjing, China). NADPH oxidase activity and superoxide anion levels were measured by enhanced lucigenin-derived chemiluminescence as we previously described [43]. A specific marker of oxidative stress, 15-F2t-isoprostane (15-F2t-IsoP), was detected by an enzyme linked immunosorbent assay (ELISA) kit (Cayman chemical, Ann Arbor, MI, USA) as described previously [44]. In similarity, an index of systemic oxidative stress, the levels of isoprostane 8-epi-prostaglandin F_2α_ (isoprostane 8-epi-PG F_2α_), was measured by using an ELISA kit (Cayman chemical, Ann Arbor, MI, USA) as described previously [45]. As a specific marker of nitrative stress, the renal nitrotyrosine levels were assayed by using a commercial kit (Millipore, Billerica, MA, USA) as depicted previously [44].

### 2.10 ELISA

The levels of tumor necrosis factor-α (TNF-α), interleukin-1β (IL-1β), vascular cellular adhesion molecule-1 (VCAM-1), and monocyte chemoattractant protein 1 (MCP-1) were evaluated by commercial ELISA kits (BOSTER, Wuhan, China) according to the manufacturer's instructions as we previously described. The protein levels of salusin-β were quantified using commercially available ELISA kits (USCN Life Science, Wuhan, China) according to the manufacturer's instructions [22,28]. The final results were normalized by protein content.

### 2.11 Quantitative real-time PCR

After extraction of total RNA from kidney tissues and cell samples using TRIzol reagent (Invitrogen), the first strand cDNA was synthesized from 1 μg of total RNAs in a 10 μL reaction using GoScript Reverse Transcription System (Promega Madison, WI, USA) following the manufacturer's instructions. The first strand cDNA served as the template for quantitative real-time PCR (qRT-PCR) was carried out in the Applied Biosystems 7500 Real Time PCR System (Applied Biosystems ABI) using GoTaq® Probe qPCR Master Mix (Promega Madison, WI, USA). The relative mRNA expression levels were calculated from the value of threshold cycle (Ct) and normalized to GAPDH by the 2^−△△CT^ method. The real-time PCR primers used in this study were shown in Supplementary Table S1 and Table S2.

### 2.12 Western blot

The collected cell samples and renal cortical tissue were lysed with RIPA lysis buffer containing phosphatase and protease inhibitors. For cell membrane or cytoplasmic protein extraction, a Membrane and Cytosol Protein Extraction Kit (Beyotime Biotechnology, Shanghai, China) was used to collect the membrane and cytoplasmic protein according to the manufacturer's protocols [46]. The protein level was determined by using BCA colorimetric protein kit (Beyotime Biotechnology, Shanghai, China). Equal amount of protein extracts were separated by SDS-PAGE, transferred onto polyvinylidene difluoride (PVDF) membranes. After blocking with 5% nonfat milk, the membranes were probed with required primary antibodies at 4 °C overnight. After incubation with horseradish peroxidase-conjugated secondary antibodies for 1 h at room temperature, the immunoblots were visualized with ECL reagents according to the manufacturer's instructions. Protein bands were normalized with β-actin/NKAα1.

### 2.13 Rac1 activity measurement

Rac1 activity was measured using a Rac1 activation assay kit (Millipore, Billerica, MA, USA). Briefly, the protein sample concentrations were measured by using the BCA Protein Assay Kit (Beyotime Institute of Biotechnology, Shanghai, China). Protein p21-activated protein kinase 1 (Pak 1) is able to bind active Rac1 form. The same total protein in each protein (1 mg) was mixed with 10 μl PAK-1 PBD (a GST fusion protein corresponding to the p21-binding domain (PBD) of human PAK-1) agarose beads for 1 h at 4 °C. Active (GTP-bound) Rac is specially combined with the p21-binding domain of p21-activated protein kinase 1. Precipitated GST bound Rac1 agarose beads were resuspended in 40 μL of 2 × reducing sample buffer and boiled for 5 min. The bound proteins were separated by 12% SDS-PAGE and immunoblotted using anti-Rac1 antibody (1:1000, Millipore, Billerica, MA, USA).

### 2.14 Statistical analysis

All results were expressed as mean ± SEM. Comparison between two groups was analyzed using Student's t-test. Comparisons for multiple groups were conducted using analysis of variance (ANOVA) followed by Bonferroni test post hoc test using the Statistical Program for Social Sciences (SPSS, version 17.0). The criterion of statistical significance was set as P value less than 0.05.

## 3 Results

### 3.1 Expressions of salusin-β in cisplatin or LPS-treated renal tubular cells and mice

**Fig. 1:**
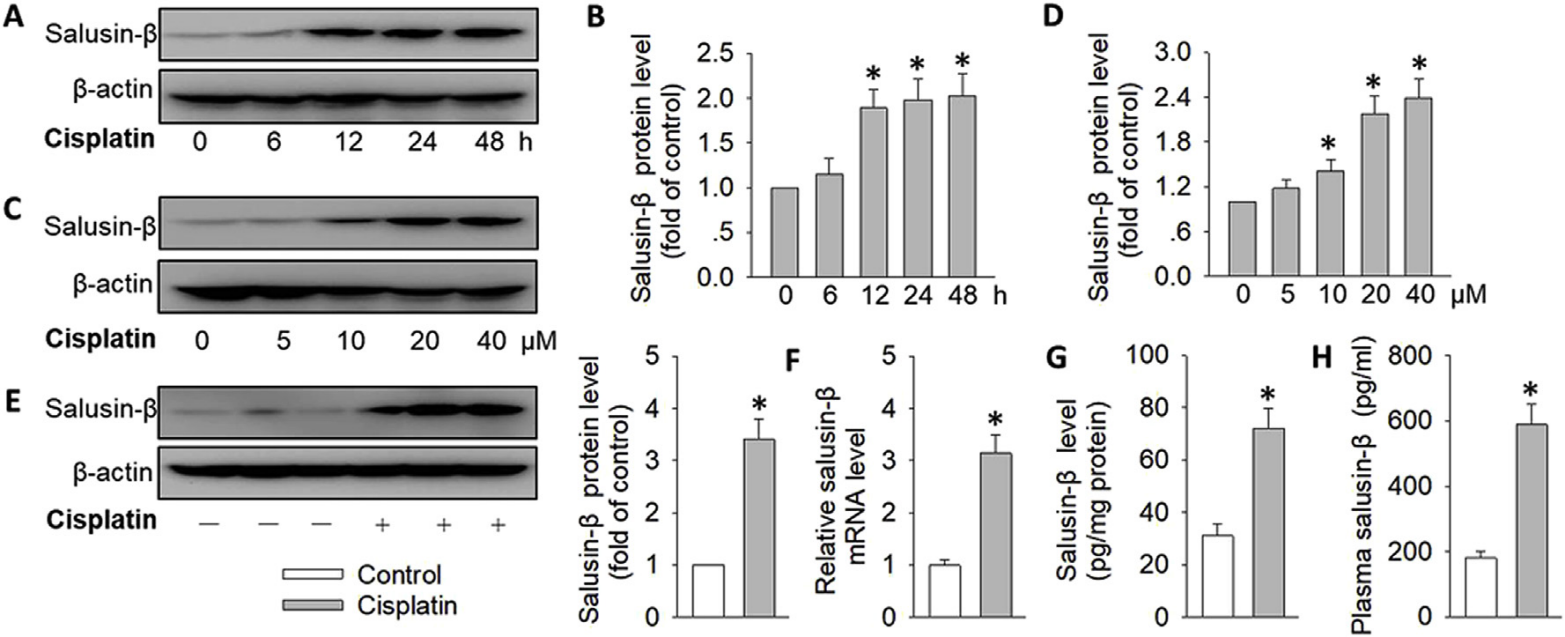
**Expressions of salusin-β in cisplatin-treated renal tubular cells and mice.** (**A**) Representative blots showing effect of cisplatin (20 μM) on the protein expression of salusin-β at 0, 6, 12, 24, 48 h. (**B**) Bar group showing the relative quantification of salusin-β. (**C**) Representative blots showing effect of cisplatin (0, 5, 10, 20, 40 μM) on the protein expression of salusin-β for 24 h. (**D**) Bar group showing the relative quantification of salusin-β. (**E**) Representative blots showing the protein expression of renal salusin-β in control mice or cisplatin-treated mice. (**F**) The mRNA expression of renal salusin-β in control mice or cisplatin-treated mice. (**G**) Renal protein levels of salusin-β in control mice or cisplatin-treated mice determined by ELISA. (**H**) Plasma salusin-β level. Values are mean ± SE. *P < 0.05 vs. 0 μM, 0 h or Control. n = 6 for each group.

During cisplatin challenge, the protein and mRNA level of salusin-β was progressively increased in a time-dependent manner when compared to controls (Fig. 1A and B&S1A). Likewise, cisplatin incubation dose-dependently upregulated the protein and mRNA level of salusin-β in renal tubular cells (Fig. 1C,D&S1B). Based on the cell results, the dose of cisplatin was selected at a dose of 20 μM for 24 h in subsequent experiments. Compared with control mice, both protein level and mRNA expressions of salusin-β are elevated in the kidney of cisplatin-induced mice (Fig. 1E and F), which was paralleled by a drastic increase in renal and plasma salusin-β level of cisplatin-treated mice (Fig. 1G and H). To examine the specific role of salusin-β in acute kidney injury, we therefore used another AKI model, LPS-induced nephropathy. Similar to the results from cisplatin-induced AKI, LPS exposure significantly incremented the expressions of salusin-β at both protein level and mRNA levels in both renal tubular cells and kidneys (Fig. S1C,D&S2). Accordingly, the dose and time frame for LPS stimulation was selected at the concentration of 10 μg/ml for 24 h during the subsequent cell experiments.

### 3.2 Effects of salusin-β knockdown or overexpression on cisplatin-induced renal tubular cell damage

**Fig. 2:**
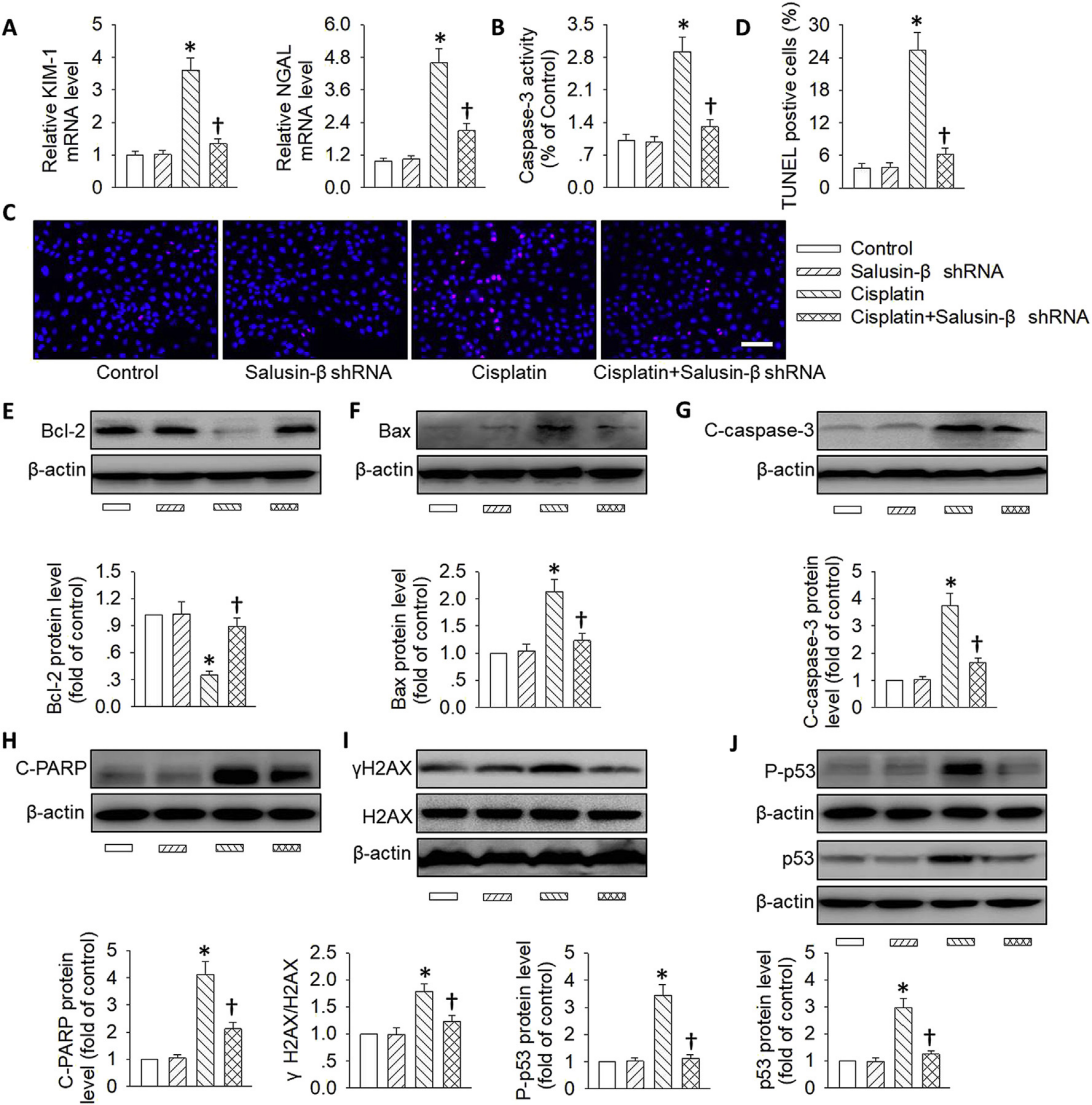
**Effects of salusin-β knockdown on cisplatin-induced renal tubular cell damage.** HK-2 cells were transfected with adenovirus mediated shRNA against salusin-β (MOI = 100) for 48 h, and then used for cisplatin (20 μM) stimulation for 24 h. (**A**) Relative mRNA levels of KIM-1 and NGAL. (**B**) Caspase-3 activity. (**C**) Cell apoptosis determined with TUNEL assay. Blue fluorescence (Hoechst 33342) shows cell nuclei and green fluorescence (TUNEL) stands for apoptotic cells. (**D**) The ratio of TUNEL-positive cells to total cells. (**E**) Representative blots and quantitative analysis of Bcl-2. (**F**) Representative blots and quantitative analysis of Bax. (**G**) Representative blots and quantitative analysis of cleaved-caspase-3 (C-caspase-3). (**H**) Representative blots and quantitative analysis of cleaved-PARP (C-PARP). (**I**) Representative blots and quantitative analysis of γH2AX at 24 h after cisplatin (20 μM) stimulation. (**J**) Representative blots and quantitative analysis of total and phosphorylated p53. Scale bar = 50 μm. Values are mean ± SE. *P < 0.05 vs. Control, †P < 0.05 vs. Cisplatin. n = 6 for each group. (For interpretation of the references to color in this figure legend, the reader is referred to the Web version of this article.)

The effects of salusin-β were first examined in renal tubular epithelial cells. Adenoviral vectors encoding salusin-β shRNA or lentivirus expressing salusin-β were used to knockdown or overexpress salusin-β. Specific knockdown or overexpression of salusin-β in tubular epithelial cells was confirmed at the mRNA and protein expressions (Fig. S1G&H). Incubation of tubular cells with cisplatin remarkably reduced cell viability and enhanced LDH release, this effect was almost completely abolished by salusin-β shRNA treatment (Fig. S3A&B). By contrast, ectopic overexpression of salusin-β further deteriorated the effect of cisplatin on cell viability and LDH release (Fig. S3C&D). Interestingly, salusin-β deficiency alone had no effect on cell viability of tubular cells, while salusin-β overexpression alone led to decreased cell viability and increased LDH release in tubular cells (Fig. S3C&D), suggesting that endogenous salusin-β overproduction may be toxic to renal tubular cells. As expected, silencing of salusin-β alleviated the effect of cisplatin on renal tubular cell injury, as demonstrated by diminished tubular cell injury markers including kidney injury molecule 1 (KIM-1) and neutrophil gelatinase-associated lipocalin (NGAL) (Fig. 2A).

Apoptotic pathway is a critical molecular mechanism that participates in cisplatin or LPS-induced nephrotoxicity, and the pro-apoptotic protein including Bax, cleaved-PARP and cleaved-caspase-3, as well as anti-apoptotic proteins including Bcl-2 are served as important players in initiating the apoptotic response [47]. Next, we examined the effects of salusin-β knockdown or overexpression on cisplatin-induced renal tubular cell apoptosis. The cisplatin-caused increases in caspase-3 activity (Fig. 2B) and TUNEL-positive cell numbers (Fig. 2C&D) were largely reduced by gene deletion of salusin-β. These findings were further supported by measurement of apoptosis-related proteins. As shown in Fig. 2E–H, cisplatin injury significantly elevated the expressions of proteins related to cell apoptosis including Bax, cleaved caspase-3 and cleaved PARP. However, the levels of anti-apoptosis related protein Bcl-2 were downregulated in response to cisplatin challenge. Moreover, we observed that cisplatin-mediated imbalance between anti-apoptotic and pro-apoptotic proteins could be reversed by gene knockdown of salusin-β (Fig. 2E–H). Apart from this, flow cytometry results further confirmed that deficiency of salusin-β markedly attenuated cisplatin-induced tubular cell apoptosis (Fig. S4).

Increased DNA damage and p53 activation are responsible for the tubular cell apoptosis in AKI models [33,48]. A critical contributor to DNA damage is p53, due to a fact that increased p53 accumulation and phosphorylation may directly induce DNA damage [49]. After incubation of cisplatin in renal tubular cells, the DNA damage marker γH2AX, total and phosphorylated p53 expressions as well as p53 targeted proapoptotic genes Puma and Bax tended to be higher, this increase was obviously antagonized by loss of salusin-β (Fig. 2I,J&S5A). Altogether, these data suggested that interference of salusin-β could prevent the apoptotic response in cisplatin-related nephrotoxicity.

**Fig. 3:**
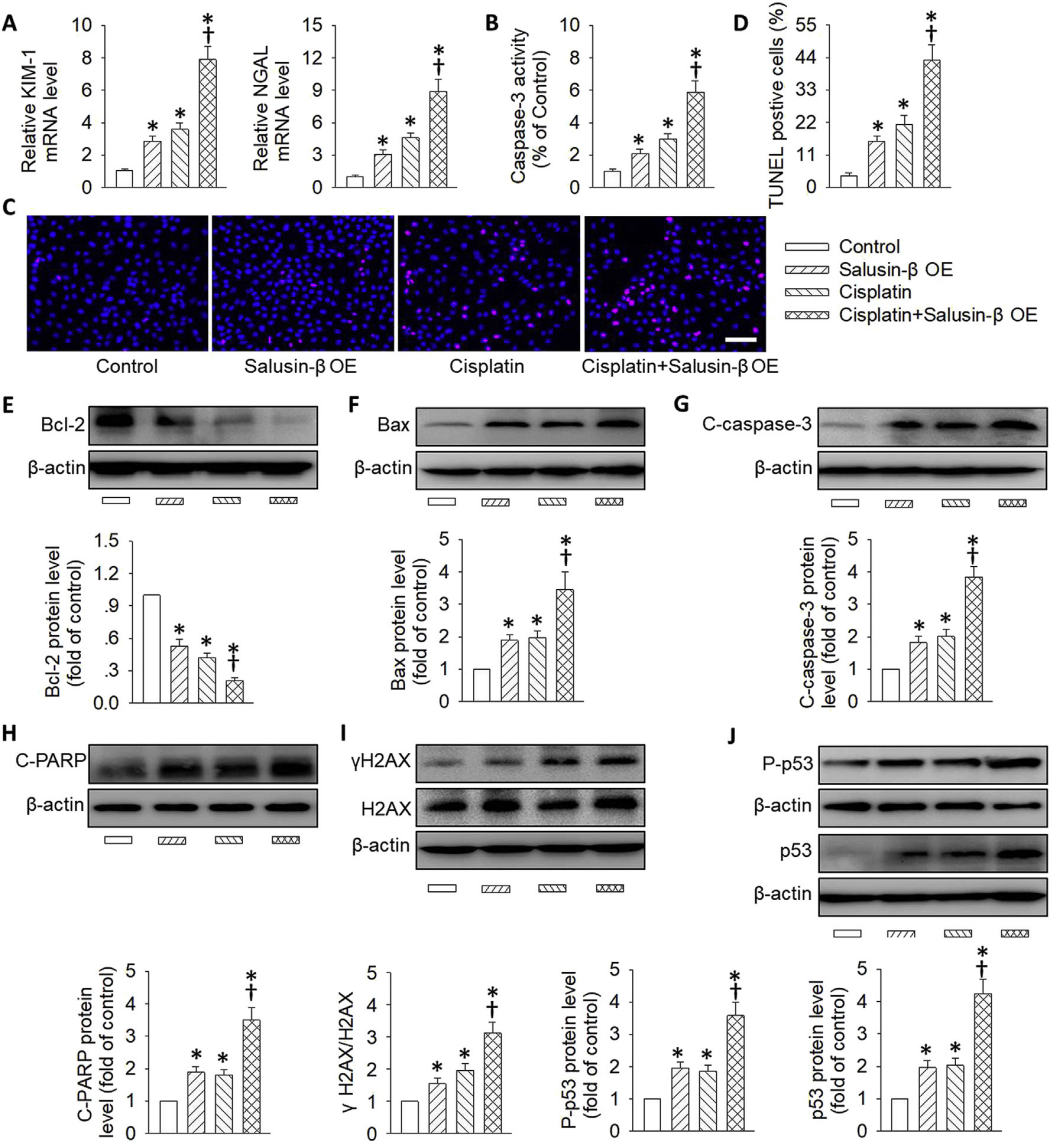
**Effects of salusin-β overexpression on cisplatin-induced renal tubular cell damage.** HK-2 cells were transfected with lentivirus expressing salusin-β (MOI = 100) for 48 h, and then used for cisplatin (20 μM) stimulation for 24 h. (**A**) Relative mRNA levels of KIM-1 and NGAL. (**B**) Caspase-3 activity. (**C**) Cell apoptosis determined with TUNEL assay. Blue fluorescence (Hoechst 33342) shows cell nuclei and green fluorescence (TUNEL) stands for apoptotic cells. (**D**) The ratio of TUNEL-positive cells to total cells. (**E**) Representative blots and quantitative analysis of Bcl-2. (**F**) Representative blots and quantitative analysis of Bax. (**G**) Representative blots and quantitative analysis of cleaved-caspase-3 (C-caspase-3). (**H**) Representative blots and quantitative analysis of cleaved-PARP (C-PARP). (**I**) Representative blots and quantitative analysis of γH2AX at 24 h after cisplatin (20 μM) stimulation. (**J**) Representative blots and quantitative analysis of total and phosphorylated p53. Scale bar = 50 μm. Values are mean ± SE. *P < 0.05 vs. Control, †P < 0.05 vs. Cisplatin. n = 6 for each group. (For interpretation of the references to color in this figure legend, the reader is referred to the Web version of this article.)

In sharp contrast, the cisplatin-induced renal tubular cell injury and DNA damage/p53 pathway-mediated cell apoptosis were further deteriorated by genetic overexpression of salusin-β (Fig. 3&S5B). In particular, the synergistic effects of salusin-β overexpression on cisplatin-induced tubular cell apoptosis were further verified by flow cytometry results (Fig. S6). Moreover, salusin-β overexpression independent of cisplatin treatment promoted renal tubular cell injury and apoptosis by regulating the DNA damage/p53 signaling pathway (Fig. 3).

### 3.3 Effects of salusin-β knockdown or overexpression on LPS-induced renal tubular cell damage

Similar to the results what we observed in cisplatin-incubated tubular cells, knockdown of salusin-β prevented, while overexpression of salusin-β intensified the decreased cell viability and increased LDH release in LPS-stimulated renal tubular cells (Fig. S7). In line with these results, the increased renal tubular cell injury markers including KIM-1 and NGAL, caspases-3 activity, TUNEL-positive cells, and the activation of DNA damage/p53 apoptotic signaling pathway were observed in LPS-challenged renal tubular cells, and these changes were obstructed by deficiency of salusin-β (Fig. S5C&S8), but were further aggravated by overexpression of salusin-β (Fig. S5D&S9). Collectively, these above results suggested that the increased apoptosis and tubular injury induced by salusin-β overexpression were mediated by the DNA damage/p53 pathway.

### 3.4 Effects of salusin-β knockdown or overexpression on cisplatin-induced oxidative stress

**Fig. 4:**
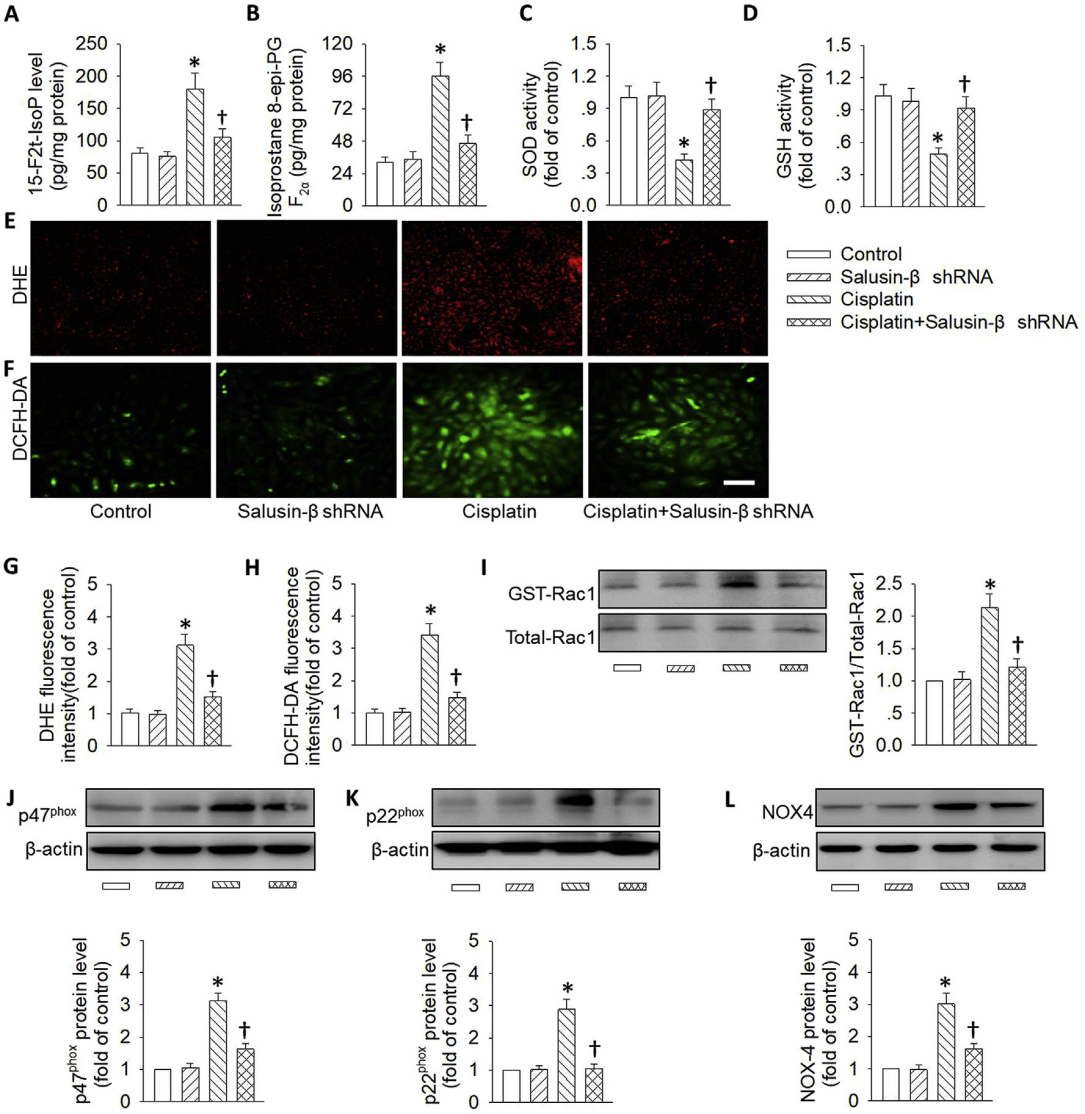
**Effects of salusin-β knockdown on cisplatin-induced renal tubular cell oxidative stress.** HK-2 cells were transfected with adenovirus mediated shRNA against salusin-β (MOI = 100) for 48 h, and then used for cisplatin (20 μM) stimulation for 24 h. (**A**) 15-F2t-isoprostane levels. (**B**) 8-iso-PGF-2α levels. (**C**) SOD activity. (**D**) GSH activity. (**E&G**) Represented images and quantitative analysis showing the levels of superoxide anions detected by DHE staining. (**F&H**) Represented images and quantitative analysis showing the levels of superoxide anions detected by DCFH-DA staining. (**I**) Representative blots and quantitative analysis of GTP-Rac1. (**J**) Representative blots and quantitative analysis of p47^phox^. (**K**) Representative blots and quantitative analysis of p22^phox^. (**L**) Representative blots and quantitative analysis of NOX-4. Scale bar = 50 μm. Values are mean ± SE. *P < 0.05 vs. Control, †P < 0.05 vs. Cisplatin. n = 6 for each group.

It is generally accepted that the development and progression of both acute and chronic kidney diseases may be primarily attributed to the imbalanced molecular mechanisms that govern oxidative stress [50]. The superoxide anions-mediated renal tubules damage may be an important contributor to the progression of AKI [51]. In light of oxidative stress, salusin-β is recently proposed as an oxidation inducer in brain tissues, VSMCs and endothelial cells in multiple disease scenarios [23,28,52]. Therefore, we wanted to determine whether salusin-β knockdown or overexpression altered cisplatin-induced oxidative stress in renal tubular cells. Upon cisplatin treatment, the massive generation of oxidative stress markers including 8-iso-PGF-2α and 15-F2t-isoprostane was observed, which was obviously suppressed by silencing of salusin-β (Fig. 4A and B). This finding was further confirmed by DHE staining and DCFH-DA staining (Fig. 4E–H). Conversely, deficiency of salusin-β restored the activities of anti-oxidases including SOD and GSH in cisplatin-treated tubular cells (Fig. 4C and D). These results indicated that the protective effect of salusin-β deficiency was at least partially due to the suppression of intracellular ROS production and restoration of anti-oxidase activities.

Rac1, a member of the Rho family of small GTPases, is a subcomponent of NADPH oxidase, which is critical for the subsequent activation of NADPH oxidase and the production of ROS [53]. Thereafter, we sought to determine whether Rac1 and NADPH oxidase subunits in renal tubular epithelial cells were activated upon stimulation with cisplatin or LPS. As expected, cisplatin treatment resulted in obvious upregulations of the NADPH oxidase subunits p47^phox^, p22^phox^, and NOX-4, as well as activated Ras-related C3 botulinum toxin substrate 1 (GTP-Rac1), the effects were enormously reduced by gene silencing of salusin-β (Fig. 4I–L).

**Fig. 5:**
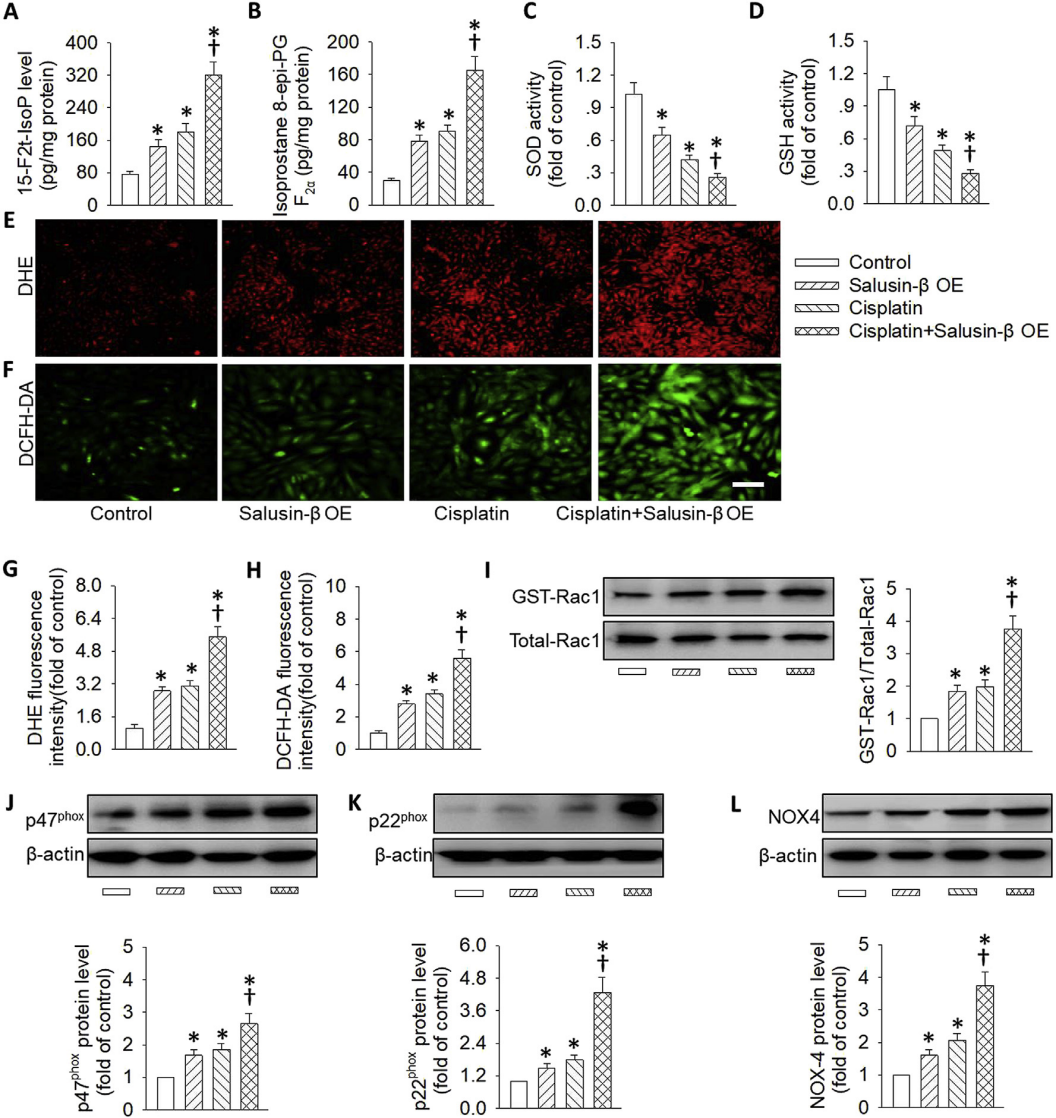
**Effects of salusin-β overexpression on cisplatin-induced renal tubular cell oxidative stress.** HK-2 cells were transfected with lentivirus expressing salusin-β (MOI = 100) for 48 h, and then used for cisplatin (20 μM) stimulation for 24 h. (**A**) 15-F2t-isoprostane levels. (**B**) 8-iso-PGF-2α levels. (**C**) SOD activity. (**D**) GSH activity. (**E&G**) Represented images and quantitative analysis showing the levels of superoxide anions detected by DHE staining. (**F&H**) Represented images and quantitative analysis showing the levels of superoxide anions detected by DCFH-DA staining. (**I**) Representative blots and quantitative analysis of GTP-Rac1. (**J**) Representative blots and quantitative analysis of p47^phox^. (**K**) Representative blots and quantitative analysis of p22^phox^. (**L**) Representative blots and quantitative analysis of NOX-4. Scale bar = 50 μm. Values are mean ± SE. *P < 0.05 vs. Control, †P < 0.05 vs. Cisplatin. n = 6 for each group.

On the contrary, the excessive ROS production, the upregulated NADPH oxidase subunits p47^phox^, p22^phox^, and NOX-4, as well as the higher Rac1 activation in renal tubular cells response to cisplatin were further exacerbated in tubular cells with salusin-β overexpression (Fig. 5). Importantly, salusin-β overexpression alone triggered renal tubular cell oxidative damage, along with increased Rac1 activity and NADPH oxidase protein expressions (Fig. 5). These present results provided supportive evidence that gene knockdown of salusin-β was capable of suppressing the NADPH oxidase-derived ROS production via inhibiting high Rac1 activity.

The translocation of p47^phox^ from the cytosol to the membrane is a critical step for excessive ROS production in diabetic kidneys [54]. Very recently, we found that overexpression of salusin-β promoted the translocation of p47^phox^ to the membrane and subsequent oxidative stress in VSMCs [55]. As a consequence, it is interesting to explore the effects of salusin-β on the membrane translocation of p47^phox^ in tubular cells. Upon cisplatin stimulation, the translocation of p47^phox^ from cytosol to the membrane increased, as evidenced by increased membrane p47^phox^ expression and decreased cytoplasmic p47phox expression in HK-2 cells (Fig. S10A). As expected, silencing of salusin-β blocked the translocation of p47phox to the membrane stimulated by cisplatin (Fig. S10A), while overexpression of salusin-β deteriorated it (Fig. S10B). These results suggest that salusin-β induces the membrane translocation of p47^phox^, thus triggering an overwhelming formation of ROS and subsequent tubular cell oxidative injury.

### 3.5 Effects of salusin-β knockdown or overexpression on LPS-induced oxidative stress

In accordance with the results from cisplatin stimulation, the active form of Rac1, Rac1-GTP, NADPH oxidase subunits p47^phox^, p22^phox^, NOX-4 protein expressions, membrane translocation of p47^phox^, and ROS overproduction were obviously upregulated in tubular cells induced by LPS, whereas salusin-β silencing markedly reversed these changes (Fig. S10A&S11). Conversely, the increased oxidative stress was further elevated in LPS-incubated tubular cells with salusin-β overexpression (Fig. S10B&S12). Together with the above results, salusin-β activated the Rac1/NAD)H oxidase/ROS signaling pathway to elicit oxidative stress, thereby leading to tubular cell injury in the absence or presence with cisplatin or LPS.

### 3.6 Role of the Rac1/NADPH oxidase/ROS pathway in salusin-β-induced tubular cell injury

**Fig. 6:**
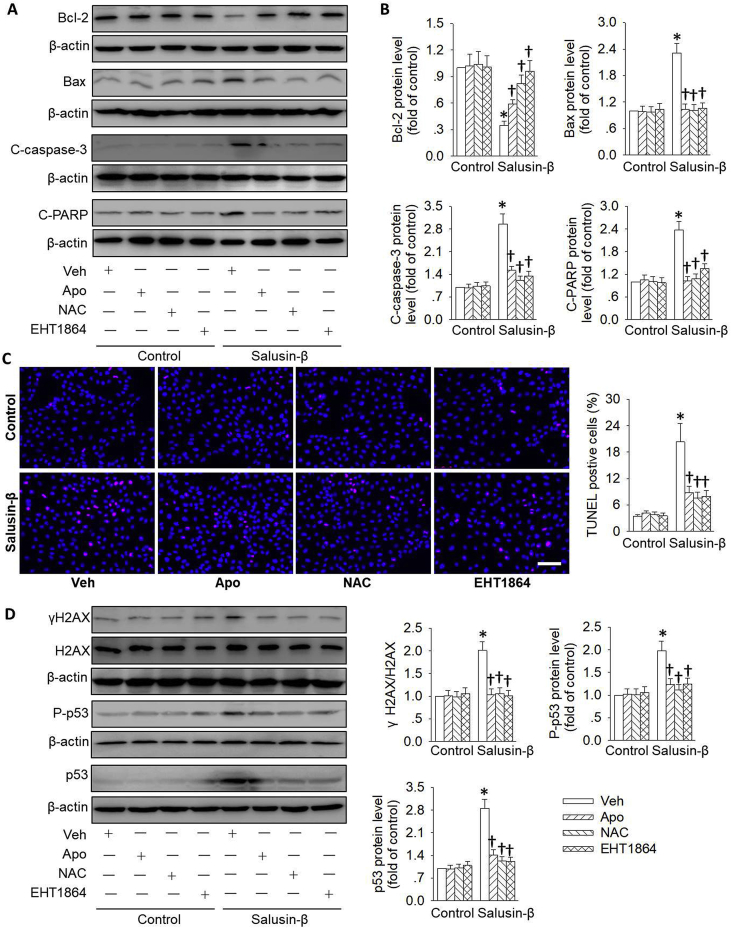
**Role of the Rac 1/NADPH oxidase/ROS pathway in salusin-β-induced tubular cell injury.** HK-2 cells were pretreated with ROS scavenger NAC (1 mM), NADPH oxidase inhibitor Apo (100 μM), and Rac-1 inhibitor EHT1864 (1 μM) for 30 min, and then transfected with lentivirus expressing salusin-β (MOI = 100) for 48 h. (**A**) Representative blots showing the protein level of Bcl-2, Bax, cleaved-caspase-3 (C-caspase-3), cleaved-PARP (C-PARP). (**B**) Quantitative analysis of the protein level of Bcl-2, Bax, cleaved-caspase-3 (C-caspase-3), cleaved-PARP (C-PARP). (**C**) Cell apoptosis determined with TUNEL assay. Blue fluorescence (Hoechst 33342) shows cell nuclei and green fluorescence (TUNEL) stands for apoptotic cells. (**D**) Representative blots and quantitative analysis of γH2AX, total and phosphorylated p53 at 48 h after salusin-β overexpression. Scale bar = 50 μm. Values are mean ± SE. *P < 0.05 vs. Control, †P < 0.05 vs. Vehicle (Veh). n = 6 for each group. (For interpretation of the references to color in this figure legend, the reader is referred to the Web version of this article.)

Given that Rac1/NADPH oxidase/ROS pathway was activated in renal tubular cells with salusin-β overexpression, we next determined whether blockade of this pathway abolished the positive effects of salusin-β overexpression on renal tubular cell apoptosis. Expectedly, pretreatment with NADPH oxidase inhibitor Apo, ROS scavenger NAC and Rac-1 inhibitor EHT1864, significantly reversed the pathological changes of apoptosis-related protein including Bcl-2, Bax, cleaved caspase-3 and cleaved PARP in renal tubular cells with salusin-β overexpression (Fig. 6A&B). In line with the attenuation of cell apoptosis protein expressions, preconditioning with Apo, NAC and EHT1864 not only interfered with salusin-β overexpression-induced ROS production (Figs. S13A–F), but also lowered renal tubular cell apoptosis (Fig. 6C) and injury (Fig. S13G&H) induced by salusin-β overexpression. Importantly enough, the augmented DNA damage marker γH2AX expression and p53 activation in salusin-β overexpressing tubular cells were erased by Apo, NAC and EHT1864, respectively (Fig. 6D).

In addition, the upregulated Bax and cleaved caspase-3 protein expressions, and enhanced TUNEL-positive cells, as well as increased renal tubular cell injury markers including KIM-1 and NGAL were normalized by the p53 inhibitor Pifithrin-α to low levels in renal tubular cells with salusin-β overexpression (Fig. S14). These results indicated that the Rac1/NADPH oxidase/ROS signaling pathway contributed to salusin-β overexpression-mediated DNA damage/p53 activation, and subsequent tubular cell injury and apoptosis.

### 3.7 Role of PKC activation in salusin-β-induced oxidative stress

**Fig. 7:**
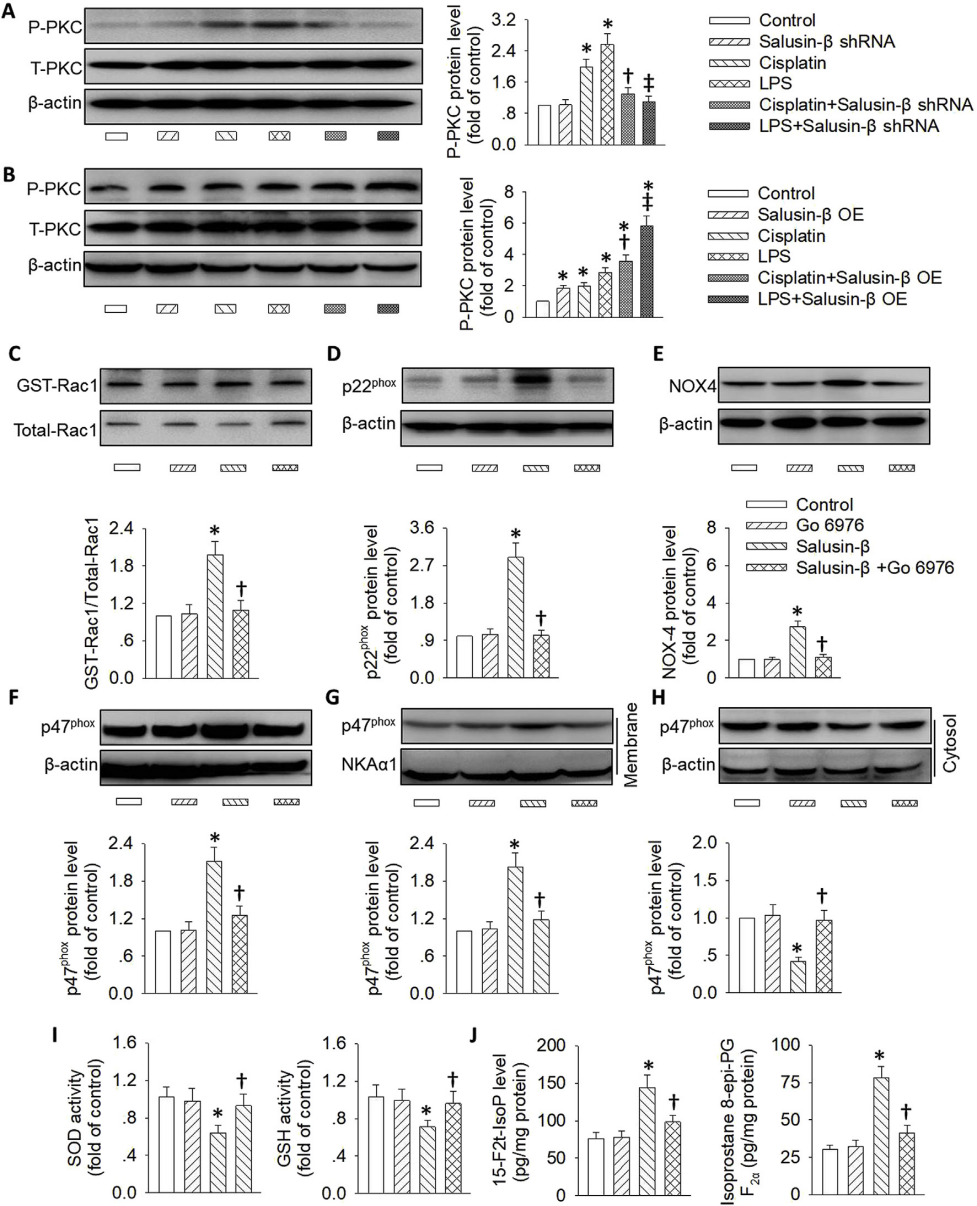
**Role of the PKC pathway in salusin-β-induced oxidative stress in renal tubular cells. (A)** Effect of salusin-β knockdown on the phosphorylated PKC in HK-2 cells treated either cisplatin or LPS. **(B)** Effect of salusin-β overexpression on the phosphorylated PKC in HK-2 cells treated either cisplatin or LPS. HK-2 cells were pretreated with PKC inhibitor Go 6976 (5 μM), and then transfected with lentivirus expressing salusin-β (MOI = 100) for 48 h. The protein expressions of GTP-Rac1 (**C**), p22^phox^ (**D**), NOX-4 (**E**), and p47^phox^ (**F**) were measured. (**G&H**) The membrane and cytosol level of p47^phox^ were also detected. (**I**) Activities of SOD and GSH. (**J**) 15-F2t-isoprostane and 8-iso-PGF-2α levels. Values are mean ± SE. *P < 0.05 vs. Control, †P < 0.05 vs. Cisplatin or Salusin-β. ‡P < 0.05 vs. LPS. n = 6 for each group.

In response to chronic or acute kidney injury, the oxidative stress is primarily attributed to protein-kinase C (PKC) activation [[56], [57], [58]]. It is reported that the PKC/NADPH oxidase/ROS signaling pathway is involved in salusin-β-induced hypertension in renovascular hypertensive rats [43]. Next, we examined whether PKC activation was responsible for salusin-β-evoked superoxide anion production in tubular cells. The phosphorylated PKC was activated in HK-2 cells treated with either cisplatin or LPS, and this effect was rectified by knockdown of salusin-β (Fig. 7A), but was intensified in HK-2 cells with salusin-β overexpression (Fig. 7B). The existing data indicated that PKC may be a downstream mediator for salusin-β in HK-2 cells. To investigate whether salusin-β-induced oxidative stress and cell apoptosis were dependent on PKC activation, we determined the effects of a PKC inhibitor, Go 6976, on salusin-β-triggered cell injury in HK-2 cells. We observed that Go 6976 pretreatment blocked the upregulated Rac1-GTP (Fig. 7C), p22^phox^ (Fig. 7D), NOX-4 (Fig. 7E) and p47^phox^ protein expressions (Fig. 7F), as well as increased membrane translocation of p47^phox^ (Fig. 7G and H) in renal tubular epithelial cells with salusin-β overexpression. The activities of SOD and GSH were downregulated (Fig. 7I), while the oxidative stress markers, 15-F2t-isoprostane and 8-iso-PGF-2α levels were augmented in HK-2 cells with salusin-β overexpression (Fig. 7J), and the above effects were also reversed by Go 6976 pretreatment. Likewise, the detrimental actions of salusin-β overexpression on tubular cell apoptosis and injury were relieved by a PKC inhibitor, Go 6976 (Fig. S15), as manifested by a restored balance between anti-apoptotic proteins and pro-apoptotic proteins (Figs. S15A–D), as well as inactivation of the DNA damage/p53 signaling pathway (Fig. S15E-L). These results provided ample evidence that salusin-β-mediated ROS/DNA damage/p53 activation and subsequent tubular cell apoptosis were dependent on activation of PKC.

### 3.8 Effects of salusin-β inhibition on renal function after AKI

**Fig. 8:**
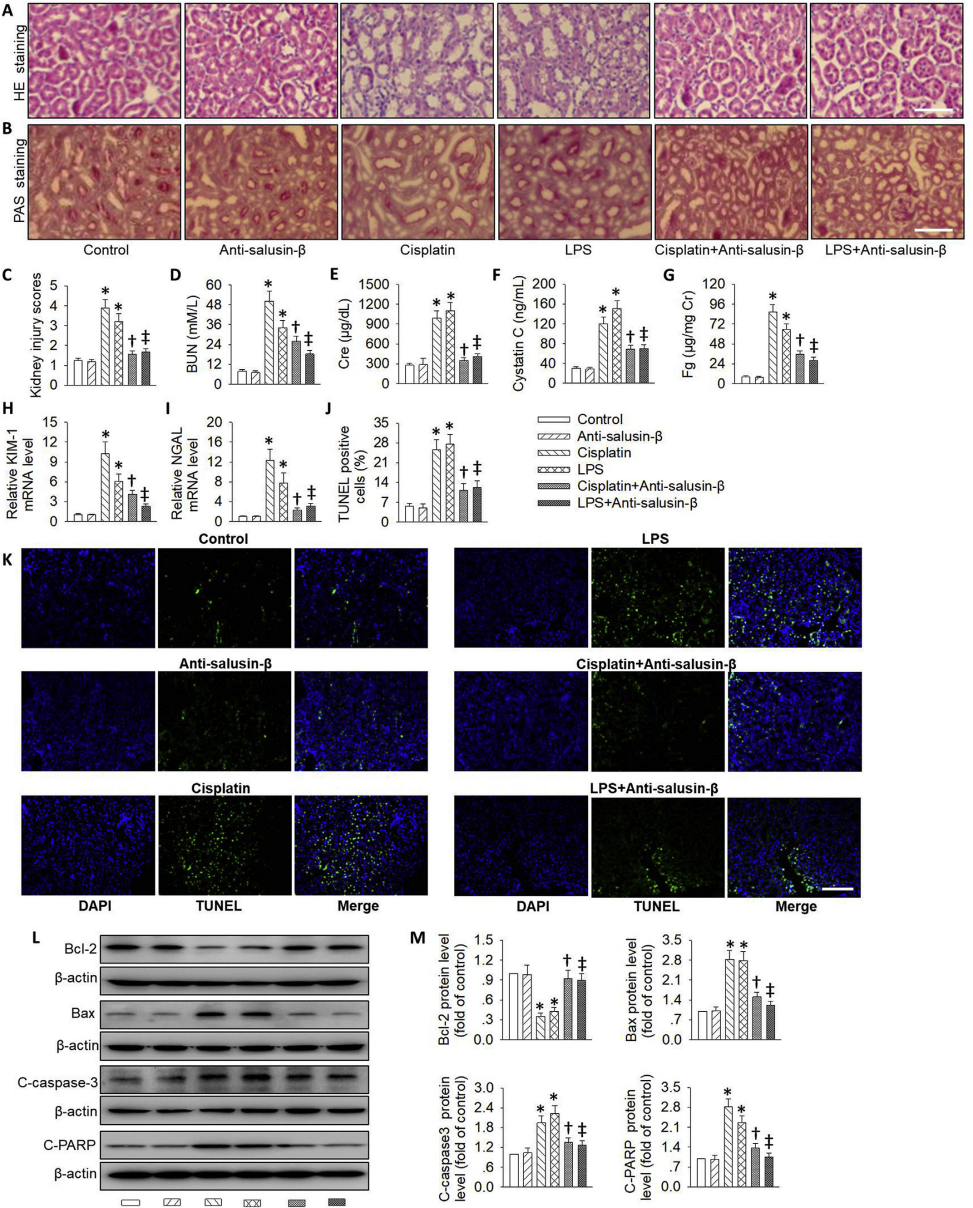
**Effects of salusin-β inhibition on renal function after AKI. (A)** HE staining of kidney sections after cisplatin or LPS-induced kidney injury. **(B)** Periodic acid-Schiff (PAS) staining of kidney sections after cisplatin or LPS-induced kidney injury. **(C)** Quantitative assessment of tubular injury. (**D**) BUN level. (**E**) Serum Cr level. (**F**) Serum cystatin C level. (**G**) Fg level. (**H**) Relative KIM-1 mRNA level. (**I**) Relative NGAL mRNA level. (**J&K**) TUNEL staining. (**L**) Representative blots showing the protein level of Bcl-2, Bax, cleaved-caspase-3 (C-caspase-3), cleaved-PARP (C-PARP). (**M**) Quantitative analysis of the protein level of Bcl-2, Bax, cleaved-caspase-3 (C-caspase-3), cleaved-PARP (C-PARP). Scale bar = 50 μm. Values are mean ± SE. *P < 0.05 vs. Control, †P < 0.05 vs. Cisplatin, ‡P < 0.05 vs. LPS. n = 6 for each group.

To investigate the effects of salusin-β inhibition on renal damage induced by cisplatin or LPS, the kidney tissues were collected after administration of cisplatin or LPS. In comparison with control kidneys, the kidneys from cisplatin or LPS treatment exhibited tubular dilatation, brush border loss and cell lysis. Strikingly, the abnormal histological lesions were significantly ameliorated in the presence of salusin-β antibody, as demonstrated by HE staining and PAS staining (Fig. 8A–C). In agreement with the histological renal morphology, neurulation of salusin-β revered the enhanced BUN, serum creatinine (Cre) and serum cystatin C in kidneys after 72 h cisplatin treatment or 24 h LPS treatment (Fig. 8D–F). To further clarify the protective role of salusin-β antibody in cisplatin or LPS-induced renal tubule injury, we examined the tubular injury markers including Fg, KIM-1 and NGAL in the kidneys. As expected, the upsurge of Fg, KIM-1 and NGAL by cisplatin or LPS was diminished with the treatment of salusin-β antibody (Fig. 8G–I). These data suggested that inhibition of salusin-β obviously mitigated cisplatin or LPS-induced renal dysfunction and pathological damages in the kidneys.

### 3.9 Effects of salusin-β inhibition on cell apoptosis, DNA damage and p53 activation after AKI

Additionally, TUNEL assay was used to detect the cell apoptosis in cisplatin or LPS-induced renal injury. More TUNEL-positive tubular cells were observed in the kidneys after cisplatin or LPS treatment, which were relieved by the neutralizing salusin-β antibody (Fig. 8J and K). We also found that the proteins levels related to cell apoptosis, such as Bax, cleaved-caspase-3 and cleaved-PARP, were upregulated in the kidneys of cisplatin or LPS-treated mice, but such protein expressions were downregulated by blockade of salusin-β (Fig. 8L&M). By contrast, a specific salusin-β blocker, anti-salusin-β antibody, restored the decreased anti-apoptotic protein Bcl-2 in response to cisplatin or LPS-evoked renal damage (Fig. 8L&M). Overall, the present data indicated that inhibition of endogenous salusin-β could lessen the apoptotic response in cisplatin or LPS-triggered nephrotoxicity.

**Fig. 9:**
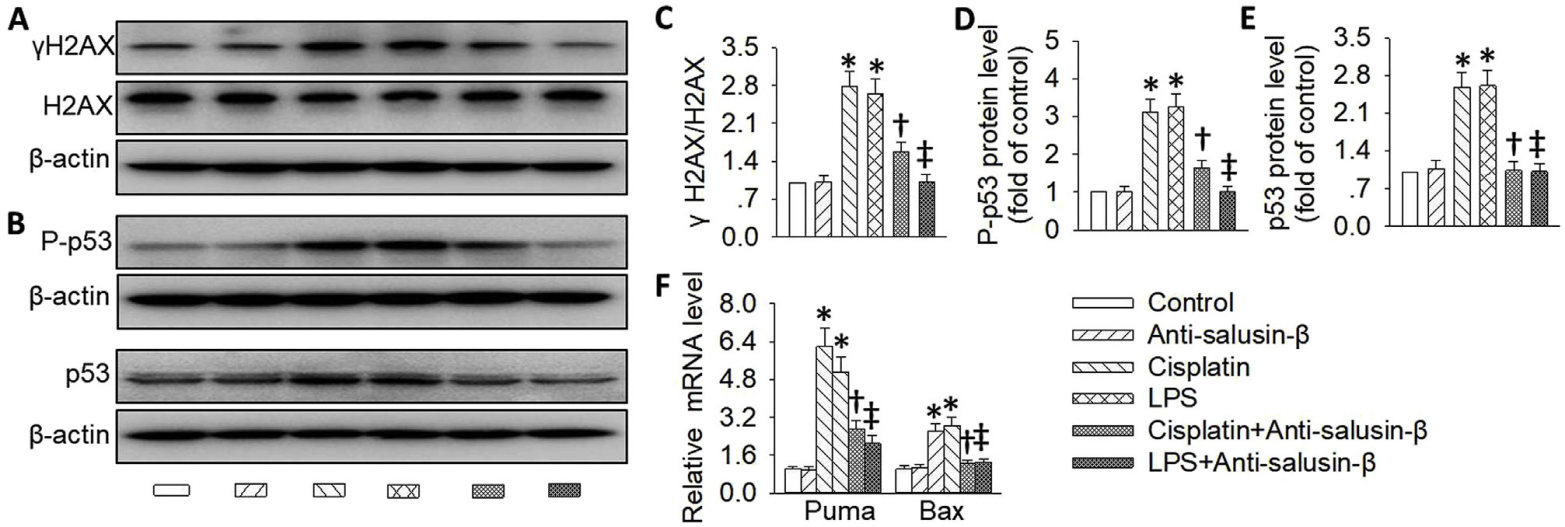
**Effects of salusin-β inhibition on DNA damage/p53 signaling after AKI. (A,C)** Representative blots and quantitative analysis of γH2AX. (**B,D,E**) Representative blots and quantitative analysis of total and phosphorylated p53. **(F)** Relative mRNA levels of p53 targeted proapoptotic genes, including Puma and Bax. Values are mean ± SE. *P < 0.05 vs. Control, †P < 0.05 vs. Cisplatin, ‡P < 0.05 vs. LPS. n = 6 for each group.

DNA damage and following p53 activation lead to cell apoptosis, which is governed by a variety of p53 targeted proapoptotic proteins, such as Puma and Bax [11]. To gain initial insights into the mechanisms by which inhibition of salusin-β protects renal tubular cells against AKI, we first determined whether the DNA damage/p53 signaling pathway was affected by salusin-β antibody. In consistence with previous reports [33,59,60], both cisplatin and LPS elicited DNA damage and p53 activation in the kidneys, as evidenced by increased expressions of γH2AX, a marker for DNA damage, p53 accumulation and phosphorylation (Fig. 9A–E), as well as p53 targeted proapoptotic genes, including Puma and Bax (Fig. 9F). However, these abnormal changes were rectified after treatment with salusin-β antibody, implying an activation of the DNA damage/p53 activation signaling pathway in AKI.

### 3.10 Effects of salusin-β inhibition on oxidative stress and inflammation after AKI

**Fig. 10:**
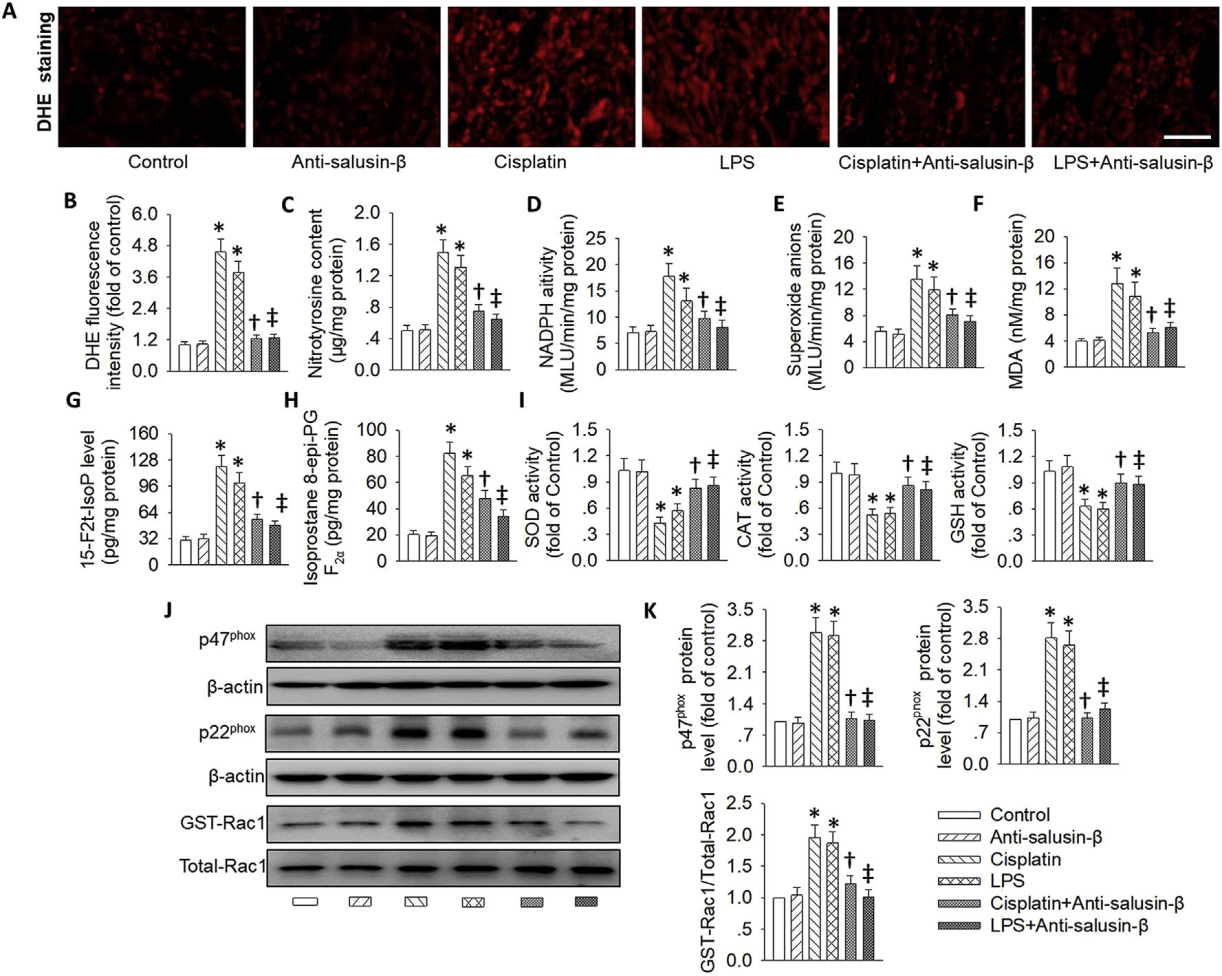
**Effect of salusin-β inhibition on oxidative stress after AKI.** (**A&B**) DHE staining. (**C**) Nitrotyrosine content. (**D**) NADPH oxidase activity. (**E**) Superoxide anions level. (**F**) MDA content. (**G**) 15-F2t-isoprostane level. (**H**) 8-iso-PGF-2α level. (**I**) Activities of SOD, CAT and GSH. (**J&K**) Representative blots and quantitative analysis of p47^phox^, p22^phox^, and GTP-Rac1. Scale bar = 50 μm. Values are mean ± SE. *P < 0.05 vs. Control, †P < 0.05 vs. Cisplatin, ‡P < 0.05 vs. LPS. n = 6 for each group.

Several studies have revealed that AKI-induced renal oxidative stress is primarily mediated by decreasing antioxidant enzyme activity, depleting intracellular concentrations of GSH [61], and potentiating the Rac1/NADPH oxidase pathway [7,62]. DHE staining results showed that anti-salusin-β antibody therapy obviously decreased the excessive ROS production in the kidneys of cisplatin or LPS-treated mice (Fig. 10A&B). The cisplatin or LPS-stimulated kidneys exhibited higher increases in nitrotyrosine content, NADPH oxidase activity, superoxide anions levels and MDA contents, whereas this rise was prevented by pretreatment with the neutralizing salusin-β antibody (Fig. 10C–F). 8-iso-PGF-2α, a marker of oxygen radical production [63], and 15-F2t-isoprostane, a specific marker of oxidative stress [64], are well-known two indicators for assessment of oxidative stress. In line with the above results, in cisplatin or LPS-induced AKI mice, we found that increased renal 8-iso-PGF-2α and 15-F2t-isoprostane production were concomitant with decreased activities of SOD, CAT and GSH in the kidneys, while these effects were again reversed by inhibition of salusin-β (Fig. 10G–I).

Moreover, the upregulated NADPH oxidase subunits p22^phox^, p47^phox^ and activated GTP-Rac1 protein levels in cisplatin or LPS-treated mice were obstructed by blockade of salusin-β (Fig. 10J and K). Also, inhibition of salusin-β reduced the translocation of p47^phox^ from cytoplasm to membrane (Figs. S16A–C) and phosphorylated PKC (Fig. S16D) in the injured kidneys. These above observations strongly suggested that cisplatin or LPS challenge induced renal oxidative stress via activation of PKC/Rac1/NADPH oxidase-dependent pathways, which were attenuated in the presence of salusin-β antibody.

Inflammatory response is also a player in the pathogenesis of renal damage induced by AKI [36,65,66]. Thus, we investigated the potential effect of salusin-β antibody on cisplatin or LPS-induced renal inflammation. Detected biochemically and histologically, the inflammatory markers including TNF-α, IL-1β, VCAM-1 and MCP-1 were all markedly raised in the kidneys of mice challenged with cisplatin or LPS, this effect was strikingly prevented by blockade of salusin-β (Fig. S17A&B). In addition, as shown by the immunohistochemistry staining, salusin-β neutralized antibody obviously blunted the upregulation of F4/80-positive macrophages in kidneys of mice treated with cisplatin or LPS (Fig. S17C), indicating an anti-inflammatory activity of the salusin-β neutralized antibody in cisplatin or LPS-induced AKI mice.

## 4 Discussion

In the present study, we investigated whether salusin-β mediated acute renal dysfunction induced by cisplatin or LPS, as well as the underlying mechanisms. Our results found that both cisplatin and LPS elevated the renal level of endogenous salusin-β, and treatment with neutralizing the salusin-β antibody ameliorated renal dysfunction and renal tubular cell apoptosis through inhibiting the PKC/ROS pathway. *In vitro* results showed that deletion of salusin-β alleviated, while overexpression of salusin-β aggravated PKC phosphorylation, oxidative stress, γH2AX-mediated DNA damage and apoptosis in renal tubular epithelial cells induced by either cisplatin or LPS. Moreover, inhibition of the PKC/ROS/DNA damage/p53 apoptotic pathway antagonized salusin-β overexpression-evoked renal tubular epithelial cell apoptosis. These results suggested that endogenous salusin-β contributed to the pathogenesis of AKI via activation of the PKC/ROS/DNA damage/p53 apoptotic pathway. However, it should be emphasized that the different mechanisms of increased oxidative stress may be present in AKI mice induced by either cisplatin or LPS. As a result, we will focus on the exact roles and mechanisms of salusin-β in one of the AKI models during our future research.

The sepsis, ischemia or nephrotoxic agents-induced AKI is a clinical disorder that is characterized by rapid and reversible kidney dysfunction [67]. It is recognized that unconscionable oxidative stress, inflammation, and renal tubular epithelial cell apoptosis are synergistically involved in the pathogenesis of AKI, and this may eventually participate in the development and progression of chronic kidney disease [10,68]. Cisplatin chemotherapy-induced nephrotoxicity and LPS-induced sepsis are taken as classical animal models of AKI [10,13]. The two AKI mouse models exhibit tubular cell apoptosis, necrosis, oxidative stress, inflammatory storm, thus leading to the renal dysfunction [36,69]. In this study, we found that salusin-β levels were upregulated in both kidneys of AKI mice and cultured renal tubular epithelial cells exposed to cisplatin or LPS. In cisplatin and LPS-induced AKI mice, the abnormal morphological changes of renal tubules and renal dysfunction markers were obviously attenuated by blockade of salusin-β in line with suppression of renal cellular apoptosis, oxidative stress and inflammation response. The increased cell apoptosis, oxidative stress and DNA damage in cisplatin or LPS-challenged renal tubular epithelial cells were eradicated by salusin-β knockdown, but exacerbated by salusin-β overexpression. Both *in vivo* and *in vitro* data demonstrated that salusin-β gene may play a critical role in the development and progression of AKI. However, the mechanisms that underlie AKI-induced salusin-β expressions in the kidneys are still unclear. It is interesting to know whether oxidative stress is involved in AKI-induced salusin-β upregulations. Based on our previous studies, we found that salusin-β expressions were not affected by ROS production as scavenging ROS had no significant effect on the expressions of salusin-β [28,55], suggesting that the expression of salusin-β is not regulated by ROS. As a consequence, the precise mechanisms by which salusin-β is overexpressed in two different AKI models warrant further studies in the future.

As no specific inhibitor or antagonist of salusin-β has been available until now, anti-salusin-β antibodies are employed to determine the roles of endogenous salusin-β in hypertension [30,70], myocardial ischemia reperfusion injury [31], and pulmonary arterial hypertension [29]. The specificity of the salusin-β staining is assessed by pre-absorption of the antibody with the full-length salusin-β, which completely abolishes salusin-β staining [71,72]. In this study, we found that intraperitoneal injection of anti-salusin-β antibodies ameliorated AKI-induced renal dysfunction in mice. It is likely that anti-salusin-β therapy has a possibility to treat AKI. However, the underlying mechanisms of anti-salusin-β antibodies in renal protective effects remain unclear. Similarly, it is unknown how they cross the blood-tissue barrier. It is noteworthy that the circulating salusin-β level was augmented in AKI mice induced by both cisplatin and LPS. Bases on this, the elevated circulating salusin-β level might be an important player in the development of AKI. We speculated that exogenous anti-salusin-β antibodies could bind to soluble circulating salusin-β, thereby attenuating the deleterious effects of redundant salusin-β on renal damage in AKI mice. Moreover, we can not exclude a possibility that the beneficial actions of anti-salusin-β antibodies on acute renal injury were dependent on their binding to renal tubular cell surface membrane protein complex. As a result, further research is required to determine the exact mechanisms of anti-salusin-β antibodies against LPS and cisplatin-induced nephrotoxicity.

It is evidenced that DNA damage could activate various cell signaling cascades for cell senescence, and/or death [73]. Recent studies have revealed that DNA damage response plays a pivotal role in renal tubular cell apoptosis induced by experimental models of AKI [74]. In mammalian system, histone H2AX phosphorylation, also called γH2AX, is a common hallmark of DNA damage [33]. The induction of γH2AX is detected in kidneys following ischemia-reperfusion or cisplatin administration, further implying the importance of DNA damage in the process of AKI [33]. It is known that p53 gene is a master player in DNA damage-induced cell apoptosis, because DNA damage could lead to p53 accumulation and phosphorylation, as well as subsequent upregulations of pro-apoptotic genes [33,75]. In line with previous reports, we found that the γH2AX levels, total and phosphorylated p53 protein levels, as well as the pro-apoptotic genes Puma and Bax were obviously boosted in renal tubular epithelial cells exposed either cisplatin or LPS. However, these effects were considerably inhibited by silencing of salusin-β. Conversely, overexpression of salusin-β further worsened these abnormal changes. In agreement with these results, the DNA damage, activation of p53 and renal cell apoptosis in the kidneys from AKI models were significantly counteracted by administration of neutralizing salusin-β antibody. All these results clearly demonstrated that salusin-β may activate the DNA damage/p53/apoptosis pathway, thus contributing to renal cell apoptosis and renal dysfunction in AKI models. However, it should bear in mind that γH2AX has a quick response to damage, and it resolves after 24 or 48 h in acute conditions when the DNA repair machinery is working well [76]. Moreover, the accumulation of high levels of γH2AX at later time points might indicate defects on DNA repair [77]. However, changes in the mechanism of DNA repair are not fully studied or discussed. The proximal tubule DNA damage response is activated when one or more of the sensor kinases detect a DNA strand break, and such sensor kinases include ataxia telangiectasia mutated (ATM), ataxia telangiectasia and Rad3-related (ATR), and DNA-dependent protein kinase (DNA-PK) [78,79]. A very recent study has established that activation of proximal tubule ATR confers protective effects against the maladaptive DNA repair and consequent renal fibrosis induced by kidney injury [80]. For this reason, it will be interesting to determine the potential roles of salusin-β in the ATM/ATR response in future studies.

Notably, salusin-β overexpression facilitated DNA damage, p53 activation and cell apoptosis in renal tubular epithelial cells even in the absence of cisplatin or LPS. Moreover, the salusin-β overexpression-induced renal tubular epithelial cell apoptosis and injury was markedly halted by a p53 inhibitor Pifithrin-α. This suggests that salusin-β overproduction was not only a critical mediator for nephrotoxic agents-induced kidney injury, but also elicited kidney injury directly. Future studies are required to investigate the abnormal expression of salusin-β under other conditions such as renal ischemia/reperfusion injury or chronic kidney disease.

Oxidative stress is defined as excessive generation of ROS and nitrogen species due to an imbalance between pro-oxidant protein expressions and antioxidant protein expressions [81]. NADPH oxidase is an enzyme complex with the function of producing superoxide anion and ROS at the expense of NADPH [82]. Of NADPH oxidase subunits, p47^phox^ plays the most important role for oxidative stress via translocating the cytosolic subunits to the membrane [82]. The massive productions of ROS and nitrogen species result in the oxidation of biological molecules such as lipids, proteins, and DNA [83]. Several studies have demonstrated that overproduction of ROS may be mediated mainly by NADPH oxidase activation and reduced activities of antioxidants including SOD, CAT and GSH, this can initiate or potentiate the development of AKI [7,69,84]. As a regulatory component of the NADPH oxidase complex, Rac1 is critically involved in the formation of ROS and progression of AKI [85,86]. Most importantly, the underlying mechanisms for AKI-induced renal tubular epithelial cell apoptosis may also involve ROS-mediated DNA damage pathway [87]. Thus, we hypothesized that ROS-induced DNA damage/p53 pathway might be a possible mechanism that underlies salusin-β overexpression-mediated renal injury. Not surprisingly, we found that the elevated membrane translocation of p47^phox^, activated Rac-1, increased NADPH oxidase-derived ROS, and decreased anti-oxidase activities in cisplatin or LPS-treated renal tubular epithelial cells were reversed by gene deficiency of salusin-β, but were deteriorated by overexpression of salusin-β. In cisplatin or LPS-challenged kidneys, inhibition of salusin-β significantly relieved oxidative damage as shown by the restoration of anti-oxidase activities, together with the blockade of ROS production and nitrogen species, as well as inactivation of Rac1 and NADPH oxidase. Noticeably, we observed that salusin-β overexpression failed to promote DNA damage/p53-mediated tubular cell apoptosis when the Rac1/NADPH oxidase/ROS axis was inhibited, suggesting that salusin-β contributed to cisplatin or LPS-caused nephrotoxicity by facilitating NADPH oxidase-derived ROS generation, followed by activation of DNA damage/p53 apoptotic cascades.

The classical PKC signaling pathway is an upstream mediator for excessive ROS production in the development of kidney injury [[56], [57], [58],^88^]. Activation of the PKC/NADPH oxidase/ROS signaling pathway plays a pathogenic role in salusin-β-induced hypertension in rats [43]. On these grounds, we hypothesized that salusin-β activated the PKC pathway to trigger ROS overproduction and subsequent oxidative damage in HK-2 cells. In the present study, we demonstrated that the phosphorylated PKC level was enhanced in HK-2 cells exposed to either cisplatin or LPS, this effect was impeded by silencing of salusin-β, but was exacerbated by overexpression of salusin-β. More importantly, the PKC inhibitor experiments supported that the PKC pathway was required for salusin-β-induced membrane translocation of p47^phox^, and activation of NADPH oxidase/DNA damage/p53 apoptotic cascades in HK-2 cells. These results suggested the involvement of the classical PKC pathway in salusin-β-mediated responses after cisplatin or LPS stimulation in HK-2 cells. However, further studies are necessary to investigate the roles of the specific PKC isoforms in the underlying mechanism of salusin-β-mediated oxidative stress in renal tubular cells.

Taken together, our findings demonstrated that salusin-β gene was responsible for cisplatin or LPS-induced nephrotoxicity by activating the PKC/ROS/DNA damage/p53 apoptotic pathway. The upregulations of phosphorylated PKC, NOX4, p47^phox^ and p22^phox^ proteins, membrane translocation of p47^phox^, activation of Rac1, reduced activities of antioxidises including SOD, CAT and GSH, were proposed as potential cellular mechanisms by which salusin-β induced oxidative stress in the process of AKI. Blockade or knockout of salusin-β may open up new fields for the prevention and treatment of AKI. However, a previous report has shown that subcutaneous administration of salusin-β reverses glomerular and tubular damage in renal ischemia/reperfusion injury, another model of AKI [89]. Contradictory to our present data, the authors demonstrated that the level of salusin-β was decreased in the serum and kidney tissues from rats with renal ischemia/reperfusion injury [89]. It seems that salusin-β may play a distinct role in different AKI models. The contradictory results may be derived from distinct AKI models, different species (rats/mouse) or administration methods of drugs (intraperitoneal/subcutaneous injection). In renal ischemia/reperfusion injury model, ischemia/hypoxia and restoration of oxygen supply eventually cause renal damage and acute renal failure [90,91]. It may be interesting to ascertain that whether salusin-β functions as an acute oxygen sensor in response to renal ischemia/reperfusion injury. The complicated results indicated that salusin-β may be a contributor to chemical/drug nephrotoxicity in mice, whereas salusin-β exhibited therapeutic potential for acute renal failure in renal ischemia/reperfusion injury rats. More research is needed to investigate exact roles of salusin-β in AKI using renal tubular epithelial cell specific salusin-β overexpression or salusin-β knockout animals.

## Declaration of competing interest

None.

## References

[1] Hoste E.A.J., Kellum J.A., Selby N.M. (2018). Global epidemiology and outcomes of acute kidney injury. Nat. Rev. Nephrol..

[2] Shum H.P., Yan W.W., Chan T.M. (2016). Recent knowledge on the pathophysiology of septic acute kidney injury: a narrative review. J. Crit. Care.

[3] Bellomo R., Kellum J.A., Ronco C., Wald R., Martensson J., Maiden M., Bagshaw S.M., Glassford N.J., Lankadeva Y., Vaara S.T., Schneider A. (2017). Acute kidney injury in sepsis. Intensive Care Med..

[4] Li Z.L., Lv L.L., Tang T.T., Wang B., Feng Y., Zhou L.T., Cao J.Y., Tang R.N., Wu M., Liu H., Crowley S.D., Liu B.C. (2019). HIF-1 alpha inducing exosomal microRNA-23a expression mediates the cross-talk between tubular epithelial cells and macrophages in tubulointerstitial inflammation. Kidney Int..

[5] Bonventre J.V., Yang L. (2011). Cellular pathophysiology of ischemic acute kidney injury. J. Clin. Investig..

[6] Waikar S.S. (2019). Precision nosology versus precision nephrology: defining acute kidney injury, again. Kidney Int..

[7] Cao X., Nie X., Xiong S., Cao L., Wu Z., Moore P.K., Bian J.S. (2018). Renal protective effect of polysulfide in cisplatin-induced nephrotoxicity. Redox Biol.

[8] Pabla N., Dong Z. (2008). Cisplatin nephrotoxicity: mechanisms and renoprotective strategies. Kidney Int..

[9] Ries F., Klastersky J. (1986). Nephrotoxicity induced by cancer chemotherapy with special emphasis on cisplatin toxicity. Am. J. Kidney Dis..

[10] Ozkok A., Edelstein C.L. (2014). Pathophysiology of cisplatin-induced acute kidney injury. BioMed Res. Int..

[11] Zhu S., Pabla N., Tang C., He L., Dong Z. (2015). DNA damage response in cisplatin-induced nephrotoxicity. Arch. Toxicol..

[12] Uchino S., Kellum J.A., Bellomo R., Doig G.S., Morimatsu H., Morgera S., Schetz M., Tan I., Bouman C., Macedo E., Gibney N., Tolwani A., Ronco C. (2005). Acute renal failure in critically ill patients: a multinational, multicenter study. Jama.

[13] Lv Y., Kim K., Sheng Y., Cho J., Qian Z., Zhao Y.Y., Hu G., Pan D., Malik A.B., Hu G. (2018). YAP controls endothelial activation and vascular inflammation through TRAF6. Circ. Res..

[14] Shichiri M., Ishimaru S., Ota T., Nishikawa T., Isogai T., Hirata Y. (2003). Salusins: newly identified bioactive peptides with hemodynamic and mitogenic activities. Nat. Med..

[15] Kolakowska U., Olanski W., Wasilewska A. (2016). Salusins in hypertension and related cardiovascular diseases. Curr. Drug Metabol..

[16] Aydin S., Aydin S. (2014). Salusin-alpha and -beta expression in heart and aorta with and without metabolic syndrome. Biotech. Histochem..

[17] Citil C., Konar V., Aydin S., Yilmaz M., Albayrak S., Ozercan I.H., Ozkan Y. (2014). Brain, liver, and serum salusin-alpha and -beta alterations in Sprague-Dawley rats with or without metabolic syndrome. Med. Sci. Monit..

[18] Sahin I., Aydin S. (2013). Serum concentration and kidney expression of salusin-alpha and salusin-beta in rats with metabolic syndrome induced by fructose. Biotech. Histochem..

[19] Fujimoto K., Hayashi A., Kamata Y., Ogawa A., Watanabe T., Ichikawa R., Iso Y., Koba S., Kobayashi Y., Koyama T., Shichiri M. (2013). Circulating levels of human salusin-beta, a potent hemodynamic and atherogenesis regulator. PLoS One.

[20] Kolakowska U., Kuroczycka-Saniutycz E., Wasilewska A., Olanski W. (2015). Is the serum level of salusin-beta associated with hypertension and atherosclerosis in the pediatric population?. Pediatr. Nephrol..

[21] Watanabe T., Nishio K., Kanome T., Matsuyama T.A., Koba S., Sakai T., Sato K., Hongo S., Nose K., Ota H., Kobayashi Y., Katagiri T., Shichiri M., Miyazaki A. (2008). Impact of salusin-alpha and -beta on human macrophage foam cell formation and coronary atherosclerosis. Circulation.

[22] Zhao M.X., Zhou B., Ling L., Xiong X.Q., Zhang F., Chen Q., Li Y.H., Kang Y.M., Zhu G.Q. (2017).

[23] Zhu X., Zhou Y., Cai W., Sun H., Qiu L. (2017). Salusin-beta mediates high glucose-induced endothelial injury via disruption of AMPK signaling pathway. Biochem. Biophys. Res. Commun..

[24] Sun H.J., Liu T.Y., Zhang F., Xiong X.Q., Wang J.J., Chen Q., Li Y.H., Kang Y.M., Zhou Y.B., Han Y., Gao X.Y., Zhu G.Q. (2015). Salusin-beta contributes to vascular remodeling associated with hypertension via promoting vascular smooth muscle cell proliferation and vascular fibrosis. Biochim. Biophys. Acta.

[25] Zhou C.H., Liu L.L., Wu Y.Q., Song Z., Xing S.H. (2012). Enhanced expression of salusin-beta contributes to progression of atherosclerosis in LDL receptor deficient mice. Can. J. Physiol. Pharmacol..

[26] Li H.B., Yu X.J., Bai J., Su Q., Wang M.L., Huo C.J., Xia W.J., Yi Q.Y., Liu K.L., Fu L.Y., Zhu G.Q., Qi J., Kang Y.M. (2019). Silencing salusin beta ameliorates heart failure in aged spontaneously hypertensive rats by ROS-relative MAPK/NF-kappaB pathways in the paraventricular nucleus. Int. J. Cardiol..

[27] Ren X.S., Ling L., Zhou B., Han Y., Zhou Y.B., Chen Q., Li Y.H., Kang Y.M., Zhu G.Q. (2017). Silencing salusin-beta attenuates cardiovascular remodeling and hypertension in spontaneously hypertensive rats. Sci. Rep..

[28] Sun H.J., Zhao M.X., Ren X.S., Liu T.Y., Chen Q., Li Y.H., Kang Y.M., Wang J.J., Zhu G.Q. (2016). Salusin-beta promotes vascular smooth muscle cell migration and intimal hyperplasia after vascular injury via ROS/NFkappaB/MMP-9 pathway. Antioxidants Redox Signal..

[29] Xu T., Zhang Z., Liu T., Zhang W., Liu J., Wang W., Wang J. (2016). Salusin-beta contributes to vascular inflammation associated with pulmonary arterial hypertension in rats. J. Thorac. Cardiovasc. Surg..

[30] Li H.B., Qin D.N., Suo Y.P., Guo J., Su Q., Miao Y.W., Sun W.Y., Yi Q.Y., Cui W., Cheng K., Zhu G.Q., Kang Y.M. (2015). Blockade of salusin-beta in hypothalamic paraventricular nucleus attenuates hypertension and cardiac hypertrophy in salt-induced hypertensive rats. J. Cardiovasc. Pharmacol..

[31] Masumura M., Watanabe R., Nagashima A., Ogawa M., Suzuki J., Shichiri M., Komuro I., Isobe M. (2013). Anti-salusin-beta antibody enhances angiogenesis after myocardial ischemia reperfusion injury. Expert Opin. Ther. Targets.

[32] Jiang Y., Liu J., Zhou Z., Liu K., Liu C. (2018). Diosmetin attenuates akt signaling pathway by modulating nuclear factor kappa-light-chain-enhancer of activated B cells (NF-kappaB)/Inducible nitric oxide synthase (iNOS) in streptozotocin (STZ)-Induced diabetic nephropathy mice. Med. Sci. Monit..

[33] Huang H., Xu C., Wang Y., Meng C., Liu W., Zhao Y., Huang X.R., You W., Feng B., Zheng Z.H., Huang Y., Lan H.Y., Qin J., Xia Y. (2018). Lethal (3) malignant brain tumor-like 2 (L3MBTL2) protein protects against kidney injury by inhibiting the DNA damage-p53-apoptosis pathway in renal tubular cells. Kidney Int..

[34] Montezano A.C., Burger D., Paravicini T.M., Chignalia A.Z., Yusuf H., Almasri M., He Y., Callera G.E., He G., Krause K.H., Lambeth D., Quinn M.T., Touyz R.M. (2010). Nicotinamide adenine dinucleotide phosphate reduced oxidase 5 (Nox 5) regulation by angiotensin II and endothelin-1 is mediated via calcium/calmodulin-dependent, rac-1-independent pathways in human endothelial cells. Circ. Res..

[35] Gu Y., Li G., Chen Y., Huang L.Y. (2016). Epac-protein kinase C alpha signaling in purinergic P2X3R-mediated hyperalgesia after inflammation. Pain.

[36] Yang Y., Yu X., Zhang Y., Ding G., Zhu C., Huang S., Jia Z., Zhang A. (2018). Hypoxia-inducible factor prolyl hydroxylase inhibitor roxadustat (FG-4592) protects against cisplatin-induced acute kidney injury. Clin. Sci. (Lond.).

[37] Zhang L., Li J., Cui L., Shang J., Tian F., Wang R., Xing G. (2018). MicroRNA-30b promotes lipopolysaccharide-induced inflammatory injury and alleviates autophagy through JNK and NF-kappaB pathways in HK-2 cells. Biomed. Pharmacother..

[38] Zhang S., Ma J., Sheng L., Zhang D., Chen X., Yang J., Wang D. (2017). Total coumarins from hydrangea paniculata show renal protective effects in lipopolysaccharide-induced acute kidney injury via anti-inflammatory and antioxidant activities. Front. Pharmacol..

[39] Li H., Feng J., Zhang Y., Feng J., Wang Q., Zhao S., Meng P., Li J. (2019). Mst1 deletion attenuates renal ischaemia-reperfusion injury: the role of microtubule cytoskeleton dynamics, mitochondrial fission and the GSK3beta-p53 signalling pathway. Redox Biol.

[40] Tang T.T., Lv L.L., Pan M.M., Wen Y., Wang B., Li Z.L., Wu M., Wang F.M., Crowley S.D., Liu B.C. (2018). Hydroxychloroquine attenuates renal ischemia/reperfusion injury by inhibiting cathepsin mediated NLRP3 inflammasome activation. Cell Death Dis..

[41] Li J., Sun K., Ma Q., Chen J., Wang L., Yang D., Chen X., Li X. (2017). Colletotrichum gloeosporioides- contaminated tea infusion blocks lipids reduction and induces kidney damage in mice. Front. Microbiol..

[42] Sun H.J., Xu D.Y., Sun Y.X., Xue T., Zhang C.X., Zhang Z.X., Lin W., Li K.X. (2017). CO-releasing molecules-2 attenuates ox-LDL-induced injury in HUVECs by ameliorating mitochondrial function and inhibiting Wnt/beta-catenin pathway. Biochem. Biophys. Res. Commun..

[43] Sun H.J., Zhang L.L., Fan Z.D., Chen D., Zhang L., Gao X.Y., Kang Y.M., Zhu G.Q. (2014). Superoxide anions involved in sympathoexcitation and pressor effects of salusin-beta in paraventricular nucleus in hypertensive rats. Acta Physiol..

[44] Su W., Zhang Y., Zhang Q., Xu J., Zhan L., Zhu Q., Lian Q., Liu H., Xia Z.Y., Xia Z. (2016). N-acetylcysteine attenuates myocardial dysfunction and postischemic injury by restoring caveolin-3/eNOS signaling in diabetic rats. Cardiovasc. Diabetol..

[45] Menini S., Amadio L., Oddi G., Ricci C., Pesce C., Pugliese F., Giorgio M., Migliaccio E., Pelicci P., Iacobini C., Pugliese G. (2006). Deletion of p66Shc longevity gene protects against experimental diabetic glomerulopathy by preventing diabetes-induced oxidative stress. Diabetes.

[46] Hou L., Bao X., Zang C., Yang H., Sun F., Che Y., Wu X., Li S., Zhang D., Wang Q. (2018). Integrin CD11b mediates alpha-synuclein-induced activation of NADPH oxidase through a Rho-dependent pathway. Redox Biol.

[47] Cummings B.S., Schnellmann R.G. (2002). Cisplatin-induced renal cell apoptosis: caspase 3-dependent and -independent pathways. J. Pharmacol. Exp. Ther..

[48] Jiang M., Wei Q., Dong G., Komatsu M., Su Y., Dong Z. (2012). Autophagy in proximal tubules protects against acute kidney injury. Kidney Int..

[49] Matt S., Hofmann T.G. (2016). The DNA damage-induced cell death response: a roadmap to kill cancer cells. Cell. Mol. Life Sci..

[50] Rajaram R.D., Dissard R., Faivre A., Ino F., Delitsikou V., Jaquet V., Cagarelli T., Lindenmeyer M., Jansen-Duerr P., Cohen C., Moll S., de Seigneux S. (2019). Tubular NOX4 expression decreases in chronic kidney disease but does not modify fibrosis evolution. Redox Biol.

[51] Mapuskar K.A., Wen H., Holanda D.G., Rastogi P., Steinbach E., Han R., Coleman M.C., Attanasio M., Riley D.P., Spitz D.R., Allen B.G., Zepeda-Orozco D. (2019). Persistent increase in mitochondrial superoxide mediates cisplatin-induced chronic kidney disease. Redox Biol.

[52] Sun H.J., Chen D., Wang P.Y., Wan M.Y., Zhang C.X., Zhang Z.X., Lin W., Zhang F. (2017). Salusin-beta is involved in diabetes mellitus-induced endothelial dysfunction via degradation of peroxisome proliferator-activated receptor gamma. Oxid Med Cell Longev.

[53] Acevedo A., Gonzalez-Billault C. (2018). Crosstalk between Rac1-mediated actin regulation and ROS production. Free Radic. Biol. Med..

[54] Park J., Kwon M.K., Huh J.Y., Choi W.J., Jeong L.S., Nagai R., Kim W.Y., Kim J., Lee G.T., Lee H.B., Ha H. (2011). Renoprotective antioxidant effect of alagebrium in experimental diabetes. Nephrol. Dial. Transplant..

[55] Sun H., Zhang F., Xu Y., Sun S., Wang H., Du Q., Gu C., Black S.M., Han Y., Tang H. (2019). Salusin-beta promotes vascular calcification via nicotinamide adenine dinucleotide phosphate/reactive oxygen species-mediated klotho downregulation. Antioxidants Redox Signal..

[56] Bicu M., Mota M., Panduru N.M., Graunteanu C., Mota E. (2010). Oxidative stress in diabetic kidney disease. Rom. J. Intern. Med..

[57] Qin T., Du R., Huang F., Yin S., Yang J., Qin S., Cao W. (2016). Sinomenine activation of Nrf 2 signaling prevents hyperactive inflammation and kidney injury in a mouse model of obstructive nephropathy. Free Radic. Biol. Med..

[58] Yang C.C., Yao C.A., Yang J.C., Chien C.T. (2014). Sialic acid rescues repurified lipopolysaccharide-induced acute renal failure via inhibiting TLR4/PKC/gp91-mediated endoplasmic reticulum stress, apoptosis, autophagy, and pyroptosis signaling. Toxicol. Sci..

[59] Wei Q., Dong G., Yang T., Megyesi J., Price P.M., Dong Z. (2007). Activation and involvement of p53 in cisplatin-induced nephrotoxicity. Am. J. Physiol. Renal. Physiol..

[60] Hammad F.T., Al-Salam S., Yuvaraju P., Lubbad L. (2018). Alda-1, an aldehyde dehydrogenase-2 agonist, causes deterioration in renal functions following ischemia-reperfusion injury due to crystalline nephropathy. Drug Dev. Res..

[61] Huang Q., Dunn R.T., Jayadev S., DiSorbo O., Pack F.D., Farr S.B., Stoll R.E., Blanchard K.T. (2001). Assessment of cisplatin-induced nephrotoxicity by microarray technology. Toxicol. Sci..

[62] Liu H., Wang L., Weng X., Chen H., Du Y., Diao C., Chen Z., Liu X. (2019). Inhibition of Brd 4 alleviates renal ischemia/reperfusion injury-induced apoptosis and endoplasmic reticulum stress by blocking FoxO4-mediated oxidative stress. Redox Biol.

[63] Delanty N., Reilly M.P., Pratico D., Lawson J.A., McCarthy J.F., Wood A.E., Ohnishi S.T., Fitzgerald D.J., FitzGerald G.A. (1997). 8-epi PGF2 alpha generation during coronary reperfusion. A potential quantitative marker of oxidant stress in vivo. Circulation.

[64] Faure P., Polge C., Monneret D., Favier A., Halimi S. (2008). Plasma 15-F2t isoprostane concentrations are increased during acute fructose loading in type 2 diabetes. Diabetes Metab..

[65] Lu M.C., Zhao J., Liu Y.T., Liu T., Tao M.M., You Q.D., Jiang Z.Y. (2019). CPUY192018, a potent inhibitor of the Keap1-Nrf 2 protein-protein interaction, alleviates renal inflammation in mice by restricting oxidative stress and NF-kappaB activation. Redox Biol.

[66] Yang T., Zhang X.M., Tarnawski L., Peleli M., Zhuge Z., Terrando N., Harris R.A., Olofsson P.S., Larsson E., Persson A.E.G., Lundberg J.O., Weitzberg E., Carlstrom M. (2017). Dietary nitrate attenuates renal ischemia-reperfusion injuries by modulation of immune responses and reduction of oxidative stress. Redox Biol.

[67] Doi K., Rabb H. (2016). Impact of acute kidney injury on distant organ function: recent findings and potential therapeutic targets. Kidney Int..

[68] Guo Y., Ni J., Chen S., Bai M., Lin J., Ding G., Zhang Y., Sun P., Jia Z., Huang S., Yang L., Zhang A. (2018). MicroRNA-709 mediates acute tubular injury through effects on mitochondrial function. J. Am. Soc. Nephrol..

[69] Chen Y., Jin S., Teng X., Hu Z., Zhang Z., Qiu X., Tian D. (2018). Hydrogen sulfide attenuates LPS-induced acute kidney injury by inhibiting inflammation and oxidative stress. Oxid Med Cell Longev.

[70] Li H.B., Qin D.N., Cheng K., Su Q., Miao Y.W., Guo J., Zhang M., Zhu G.Q., Kang Y.M. (2015). Central blockade of salusin beta attenuates hypertension and hypothalamic inflammation in spontaneously hypertensive rats. Sci. Rep..

[71] Takenoya F., Hori T., Kageyama H., Funahashi H., Takeuchi M., Kitamura Y., Shichiri M., Shioda S. (2005). Coexistence of salusin and vasopressin in the rat hypothalamo-hypophyseal system. Neurosci. Lett..

[72] Chen W.W., Sun H.J., Zhang F., Zhou Y.B., Xiong X.Q., Wang J.J., Zhu G.Q. (2013). Salusin-beta in paraventricular nucleus increases blood pressure and sympathetic outflow via vasopressin in hypertensive rats. Cardiovasc. Res..

[73] Yan M., Tang C., Ma Z., Huang S., Dong Z. (2016). DNA damage response in nephrotoxic and ischemic kidney injury. Toxicol. Appl. Pharmacol..

[74] Ma Z., Wei Q., Dong G., Huo Y., Dong Z. (2014). DNA damage response in renal ischemia-reperfusion and ATP-depletion injury of renal tubular cells. Biochim. Biophys. Acta.

[75] Beckerman R., Prives C. (2010). Transcriptional regulation by p53. Cold Spring Harb Perspect Biol.

[76] Molitoris B.A. (2019). DNA damage response protects against progressive kidney disease. J. Clin. Investig..

[77] Anglada T., Repulles J., Espinal A., LaBarge M.A., Stampfer M.R., Genesca A., Martin M. (2019). Delayed gammaH2AX foci disappearance in mammary epithelial cells from aged women reveals an age-associated DNA repair defect. Aging (N Y).

[78] Blackford A.N., Jackson S.P. (2017). ATM, ATR, and DNA-PK: the trinity at the heart of the DNA damage response. Mol. Cell.

[79] Marechal A., Zou L. (2013). DNA damage sensing by the ATM and ATR kinases. Cold Spring Harb Perspect Biol.

[80] Kishi S., Brooks C.R., Taguchi K., Ichimura T., Mori Y., Akinfolarin A., Gupta N., Galichon P., Elias B.C., Suzuki T., Wang Q., Gewin L., Morizane R., Bonventre J.V. (2019). Proximal tubule ATR regulates DNA repair to prevent maladaptive renal injury responses. J. Clin. Investig..

[81] Daenen K., Andries A., Mekahli D., Van Schepdael A., Jouret F., Bammens B. (2019). Oxidative stress in chronic kidney disease. Pediatr. Nephrol..

[82] Rastogi R., Geng X., Li F., Ding Y. (2016). NOX activation by subunit interaction and underlying mechanisms in disease. Front. Cell. Neurosci..

[83] Ratliff B.B., Abdulmahdi W., Pawar R., Wolin M.S. (2016). Oxidant mechanisms in renal injury and disease. Antioxidants Redox Signal..

[84] Meng X.M., Ren G.L., Gao L., Yang Q., Li H.D., Wu W.F., Huang C., Zhang L., Lv X.W., Li J. (2018). NADPH oxidase 4 promotes cisplatin-induced acute kidney injury via ROS-mediated programmed cell death and inflammation. Lab. Investig..

[85] Heasman S.J., Ridley A.J. (2008). Mammalian Rho GTPases: new insights into their functions from in vivo studies. Nat. Rev. Mol. Cell Biol..

[86] Gao G., Wang W., Tadagavadi R.K., Briley N.E., Love M.I., Miller B.A., Reeves W.B. (2014). TRPM2 mediates ischemic kidney injury and oxidant stress through RAC1. J. Clin. Investig..

[87] Moonen L., D'Haese P.C., Vervaet B.A. (2018). Epithelial cell cycle behaviour in the injured kidney. Int. J. Mol. Sci..

[88] Noh H., King G.L. (2007). The role of protein kinase C activation in diabetic nephropathy. Kidney Int. Suppl..

[89] Cakir M., Duzova H., Taslidere A., Orhan G., Ozyalin F. (2017). Protective effects of salusin-alpha and salusin-beta on renal ischemia/reperfusion damage and their levels in ischemic acute renal failure. Biotech. Histochem..

[90] Thuillier R., Hauet T. (2012). Role of translocator protein in renal ischemia reperfusion, renal preservation and acute kidney injury. Curr. Mol. Med..

[91] Dobashi K., Ghosh B., Orak J.K., Singh I., Singh A.K. (2000). Kidney ischemia-reperfusion: modulation of antioxidant defenses. Mol. Cell. Biochem..

