# Supplementary material for: Salusin-β mediates tubular cell apoptosis in acute kidney injury: Involvement of the PKC/ROS signaling pathway
Source: Redox Biol. 2019 Dec 20;30:101411. doi: 10.1016/j.redox.2019.101411 (PMC6939056; doi:10.1016/j.redox.2019.101411)
Supplement: Multimedia component 1 [file mmc1.docx]

**Supplementary Materials**

(17 supplementary figures and 2 supplementary tables)

**Salusin-β mediates tubular cell apoptosis in acute kidney injury: involvement of the PKC/ROS signaling pathway**

**
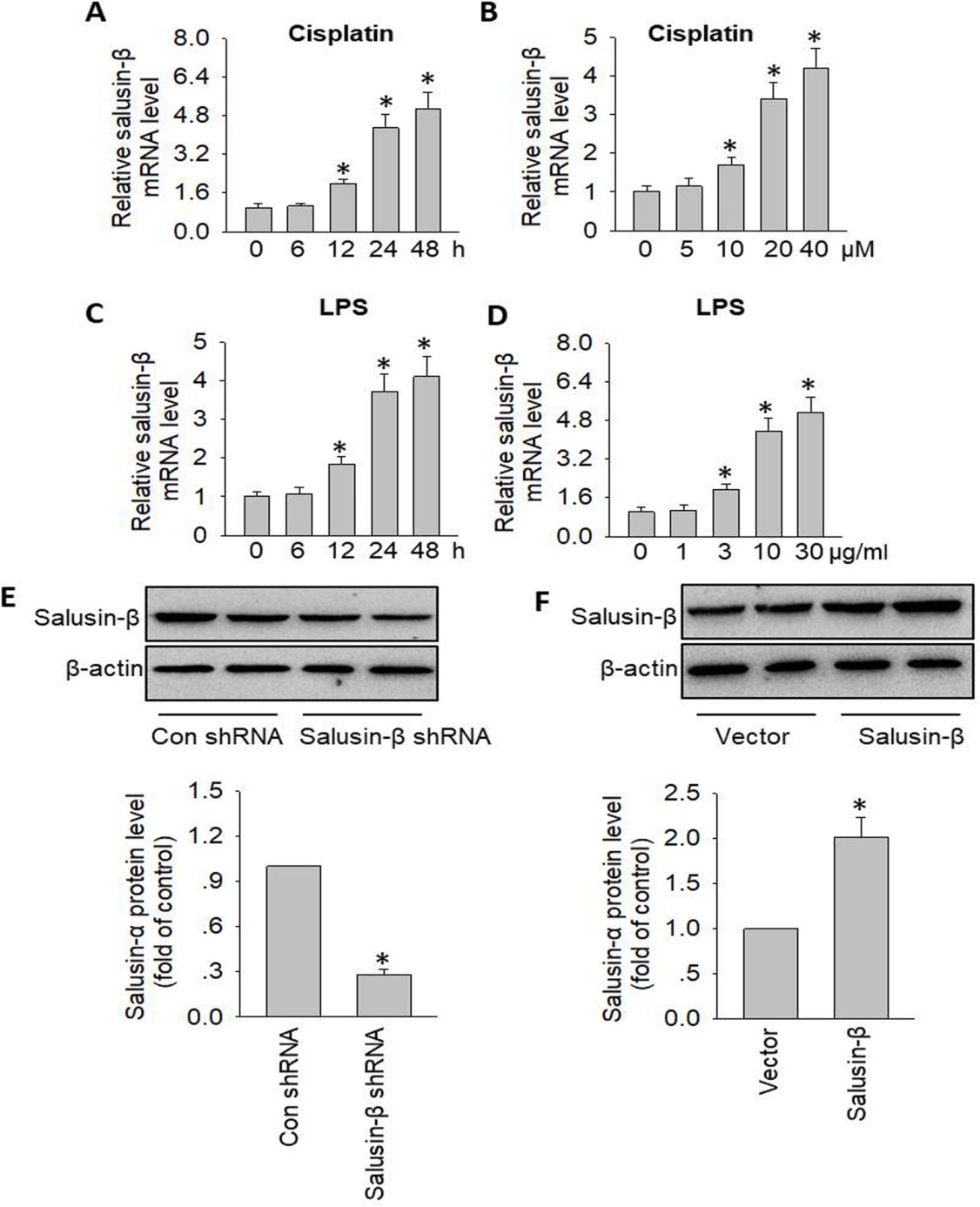
**

**Fig. S1**. **Expressions of salusin-β in renal tubular cells.** (**A**) Effect of cisplatin (20 μM) on the mRNA expression of salusin-β at 0, 6, 12, 24, 48 h. (**B**) Effect of cisplatin (0, 5, 10, 20, 40 μM) on the mRNA expression of salusin-β for 24 h. (**C**) Effect of LPS (10 μg/ml) on the protein expression of salusin-β at 0, 6, 12, 24, 48 h. (**D**) Effect of LPS (0, 1, 3, 10, 30 μg/ml) on the protein expression of salusin-β for 24 h. (**E**) The protein expression of salusin-β after salusin-β knockdown. (**F**) The protein expression of salusin-β after salusin-β overexpression. Values are mean±SE. * P < 0.05 vs. 0 μM, 0 h, 0 μM, Control (Con) shRNA, or Vector. n = 6 for each group.


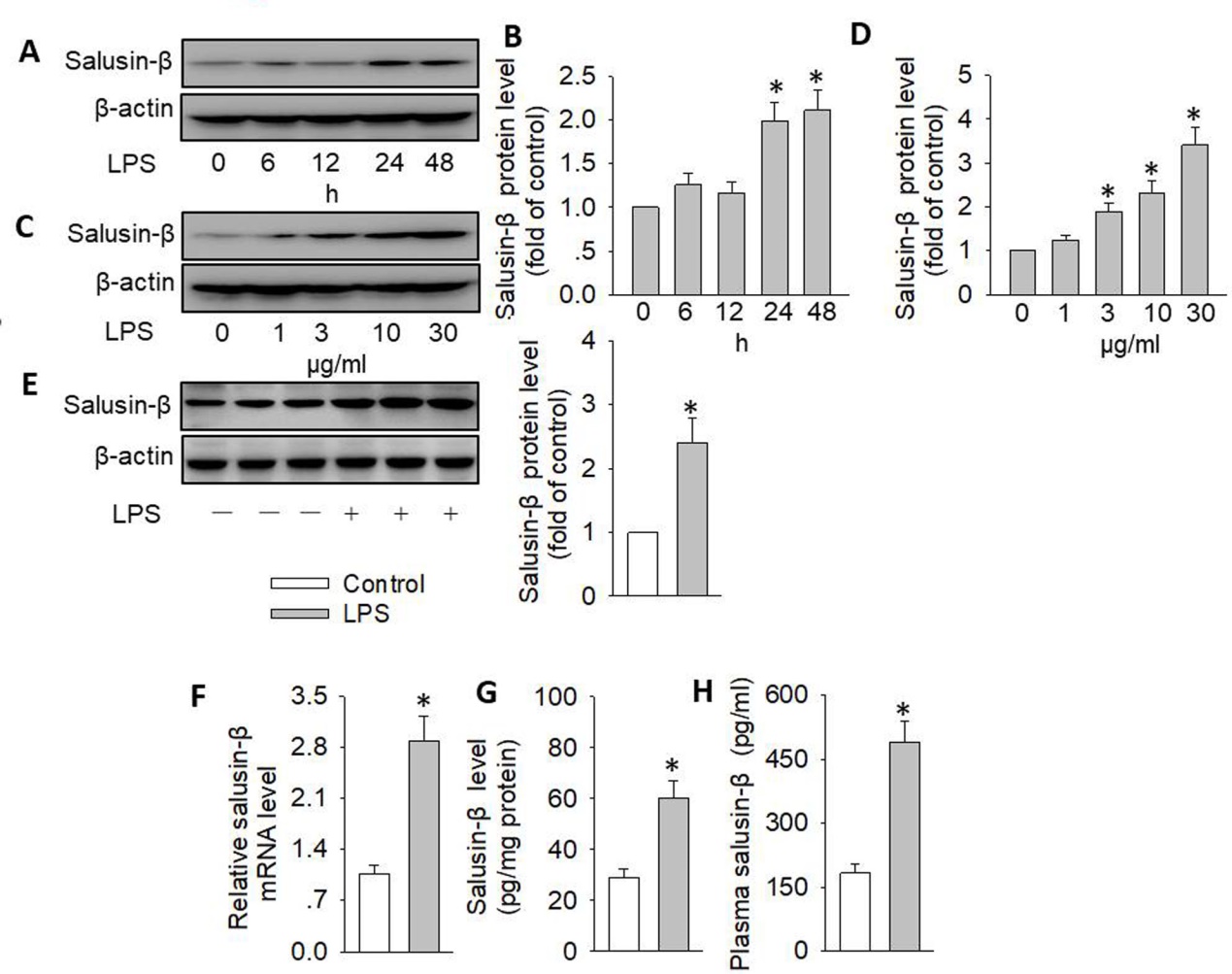


**Fig. S2. Expressions of salusin-β in LPS-treated renal tubular cells and mice.** (**A**) Representative blots showing effect of **LPS** (10 μg/ml) on the protein expression of salusin-β at 0, 6, 12, 24, 48 h. (**B**) Bar group showing the relative quantification of salusin-β. (**C**) Representative blots showing effect of **LPS** (0, 1, 3, 10, 30 μg/ml) on the protein expression of salusin-β for 24 h. (**D**) Bar group showing the relative quantification of salusin-β. (**E**) Representative blots showing the protein expression of renal salusin-β in control mice or calcium LPS-treated mice. (**F**) The mRNA expression of renal salusin-β in control mice or calcium LPS-treated mice. (**G**) Renal protein levels of salusin-β in control mice or LPS-treated mice determined by ELISA. (**H**) Plasma salusin-β level. Values are mean±SE. * P < 0.05 vs. 0 mM, 0 h or Control. n = 6 for each group.


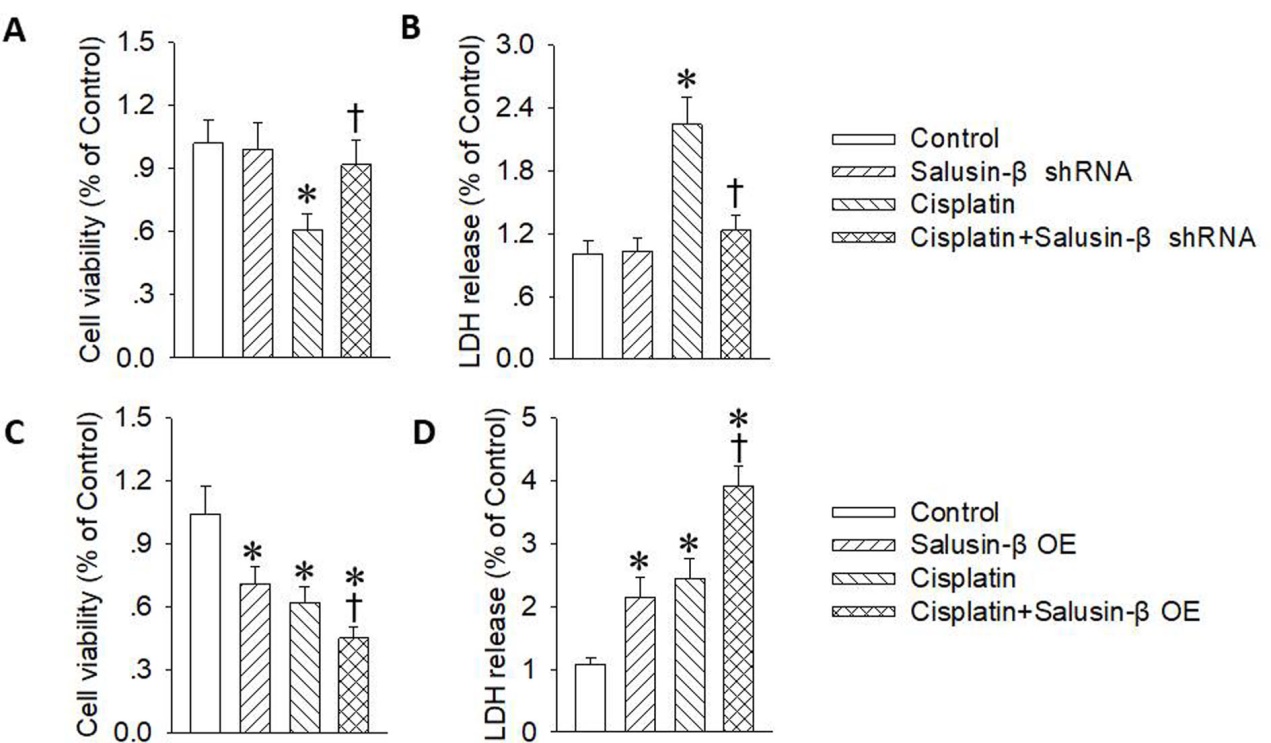


**Fig. S3**. **Cell viability and LDH release determination**. Effect of salusin-β knockdown on cisplatin-induced renal tubular cell viability (**A**) and LDH release (**B**). Effect of salusin-β overexpression on cisplatin-induced renal tubular cell viability (**C**) and LDH release (**D**). Values are mean±SE. * P < 0.05 vs. Control, † P < 0.05 vs. Cisplatin. n = 6 for each group.


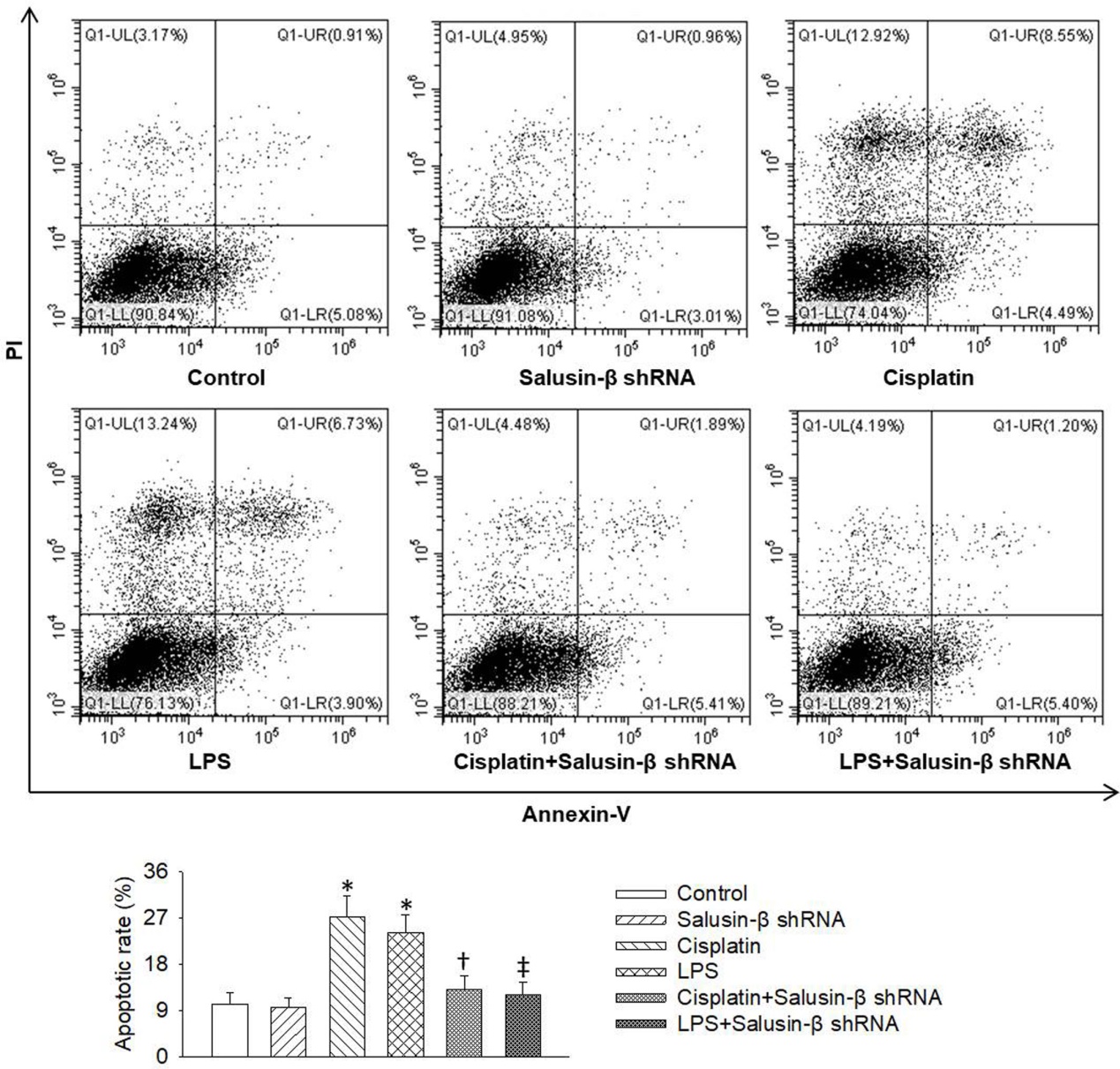


**Fig. S4**. **Effects of salusin-β deficiency on cell apoptosis induced by cisplatin or LPS**. The cell apoptosis was measured by flow cytometry. Values are mean±SE. * P < 0.05 vs. Control, † P < 0.05 vs. Cisplatin. ‡ P < 0.05 vs. LPS. n = 6 for each group.

**
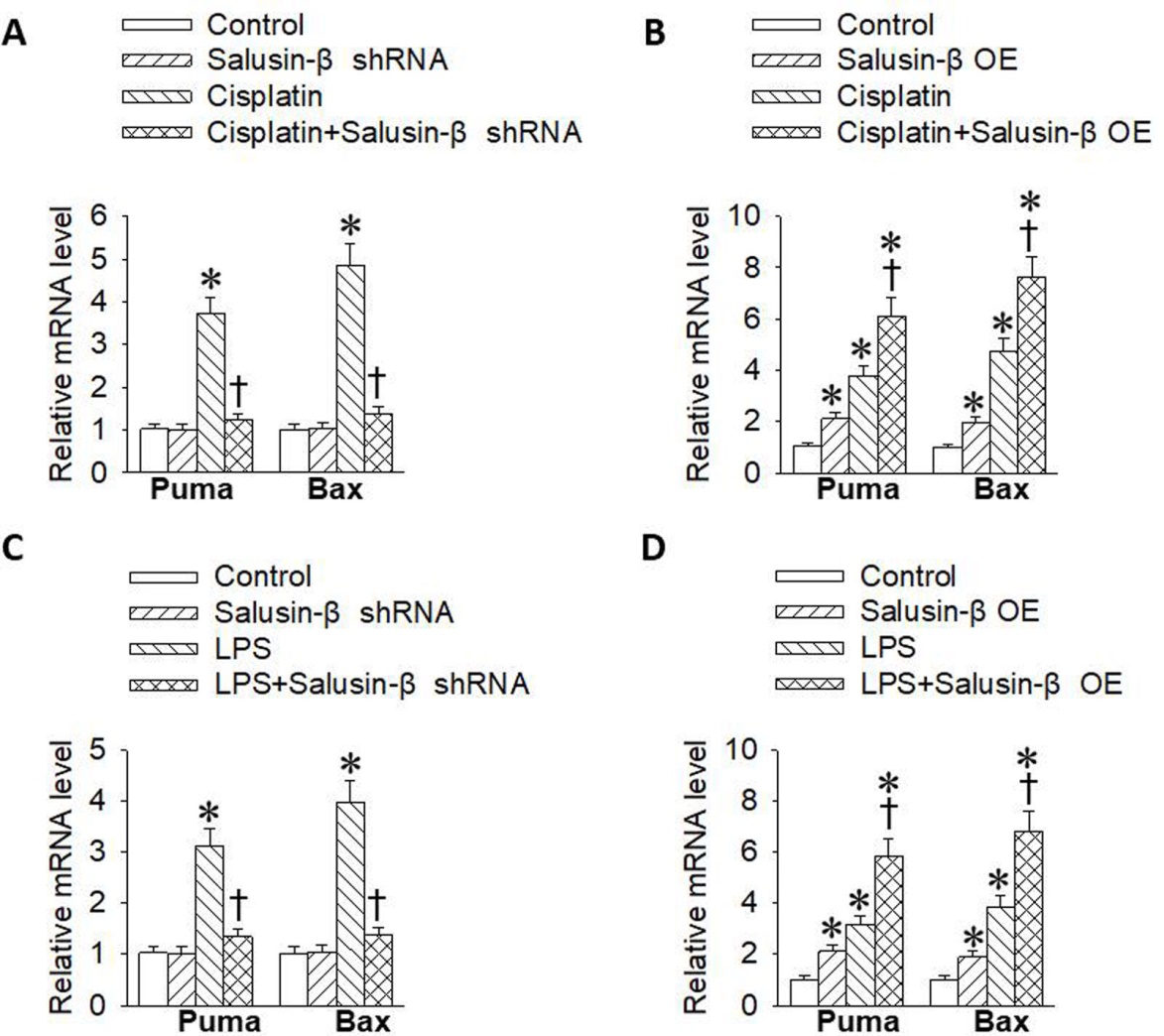
**

**Fig. S5**. **The mRNA levels of Puma and Bax.** Effect of salusin-β knockdown (**A**) or salusin-β overexpression (**B**) on cisplatin-induced expressions of Puma and Bax. Effect of salusin-β knockdown (**C**) or salusin-β overexpression (**D**) on LPS-induced expressions of Puma and Bax. Values are mean±SE. * P < 0.05 vs. Control, † P < 0.05 vs. Cisplatin or LPS. n = 6 for each group.


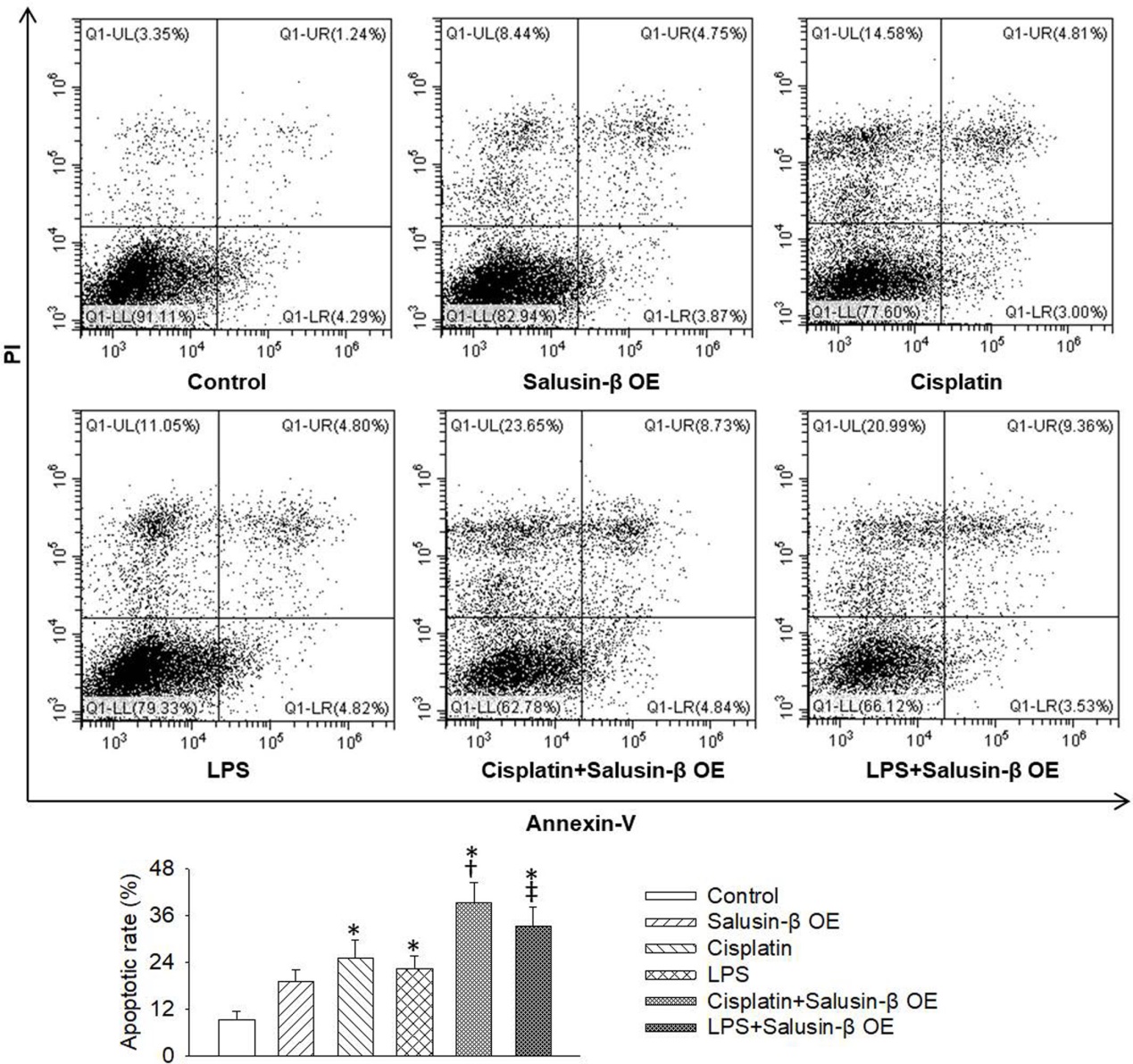


**Fig. S6**. **Effects of salusin-β overexpression on cell apoptosis induced by cisplatin or LPS**. The cell apoptosis was measured by flow cytometry. Values are mean±SE. * P < 0.05 vs. Control, † P < 0.05 vs. Cisplatin. ‡ P < 0.05 vs. LPS. n = 6 for each group.

**
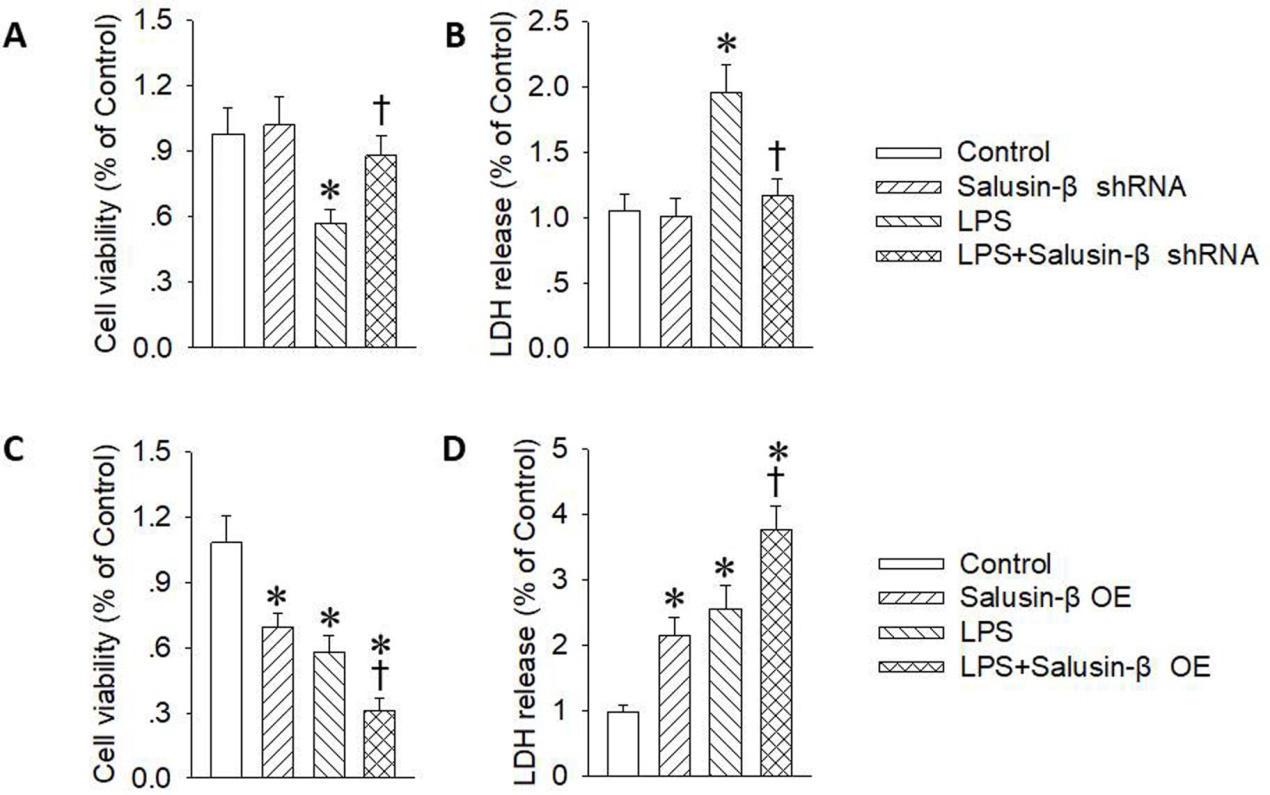
**

**Fig. S7**. **Cell viability and LDH release determination**. Effect of salusin-β knockdown on LPS-induced renal tubular cell viability (**A**) and LDH release (**B**). Effect of salusin-β overexpression on LPS-induced renal tubular cell viability (**C**) and LDH release (**D**). Values are mean±SE. * P < 0.05 vs. Control, † P < 0.05 vs. LPS. n = 6 for each group.


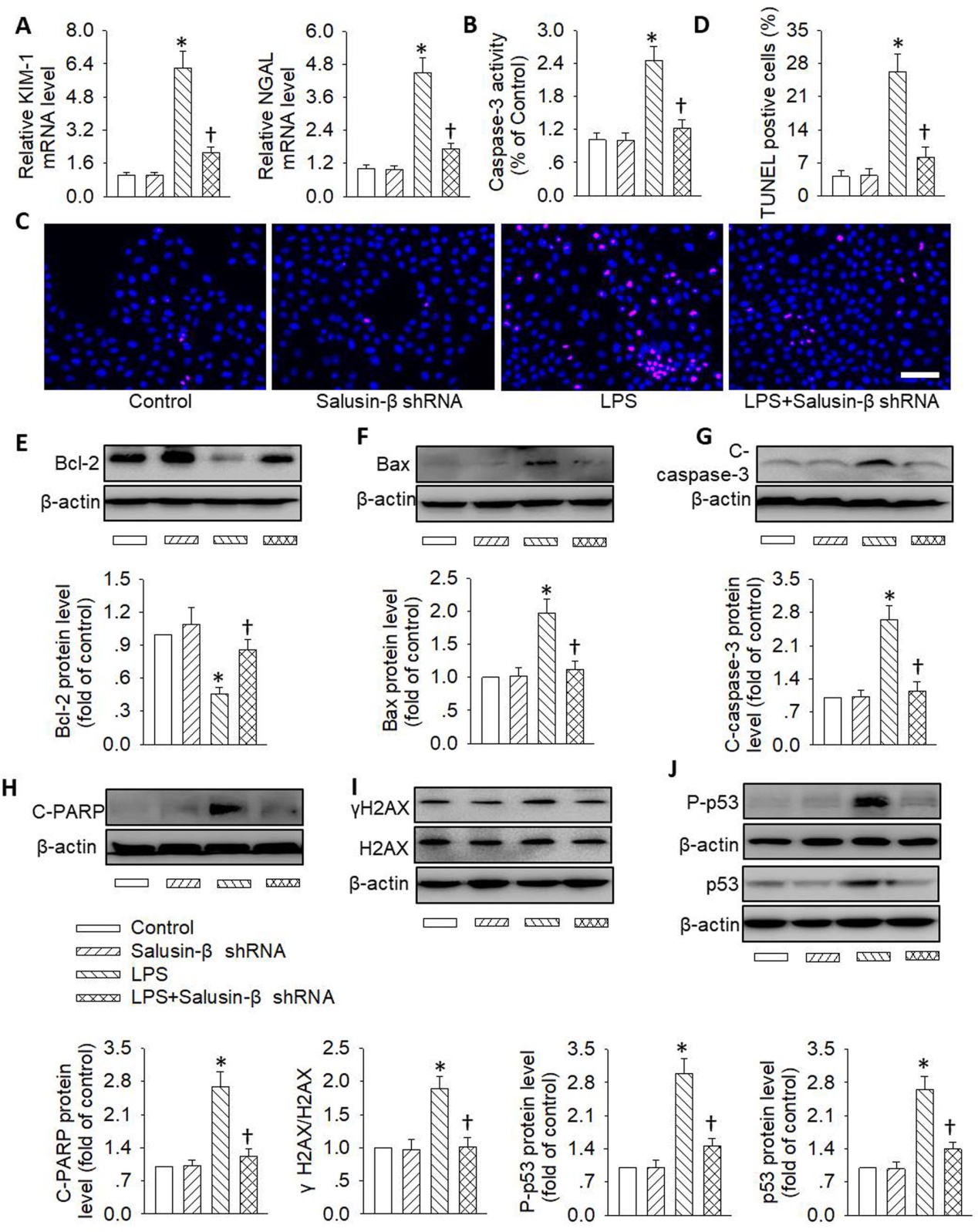


**Fig. S8**. **Effect of salusin-β knockdown on LPS-induced renal tubular cell damage.** HK-2 cells were transfected with adenovirus mediated shRNA against salusin-β (MOI = 100) for 48 h, and then used for LPS (10 μg/ml) stimulation for 24 h. (**A**) Relative mRNA levels of KIM-1 and NGAL. (**B**) Caspase-3 activity. (**C**) Cell apoptosis determined with TUNEL assay. Blue fluorescence (Hoechst 33342) shows cell nuclei and green fluorescence (TUNEL) stands for apoptotic cells. (**D**) The ratio of TUNEL-positive cells to total cells. (**E**) Representative blots and quantitative analysis of Bcl-2. (**F**) Representative blots and quantitative analysis of Bax. (**G**) Representative blots and quantitative analysis of cleaved-caspase-3 (C-caspase-3). (**H**) Representative blots and quantitative analysis of cleaved-PARP (C-PARP). (**I**) Representative blots and quantitative analysis of γH2AX at 24 h after LPS (10 μg/ml) stimulation. (**J**) Representative blots and quantitative analysis of total and phosphorylated p53. Scale bar=50 μm. Values are mean±SE. * P < 0.05 vs. Control, † P < 0.05 vs. LPS. n = 6 for each group.


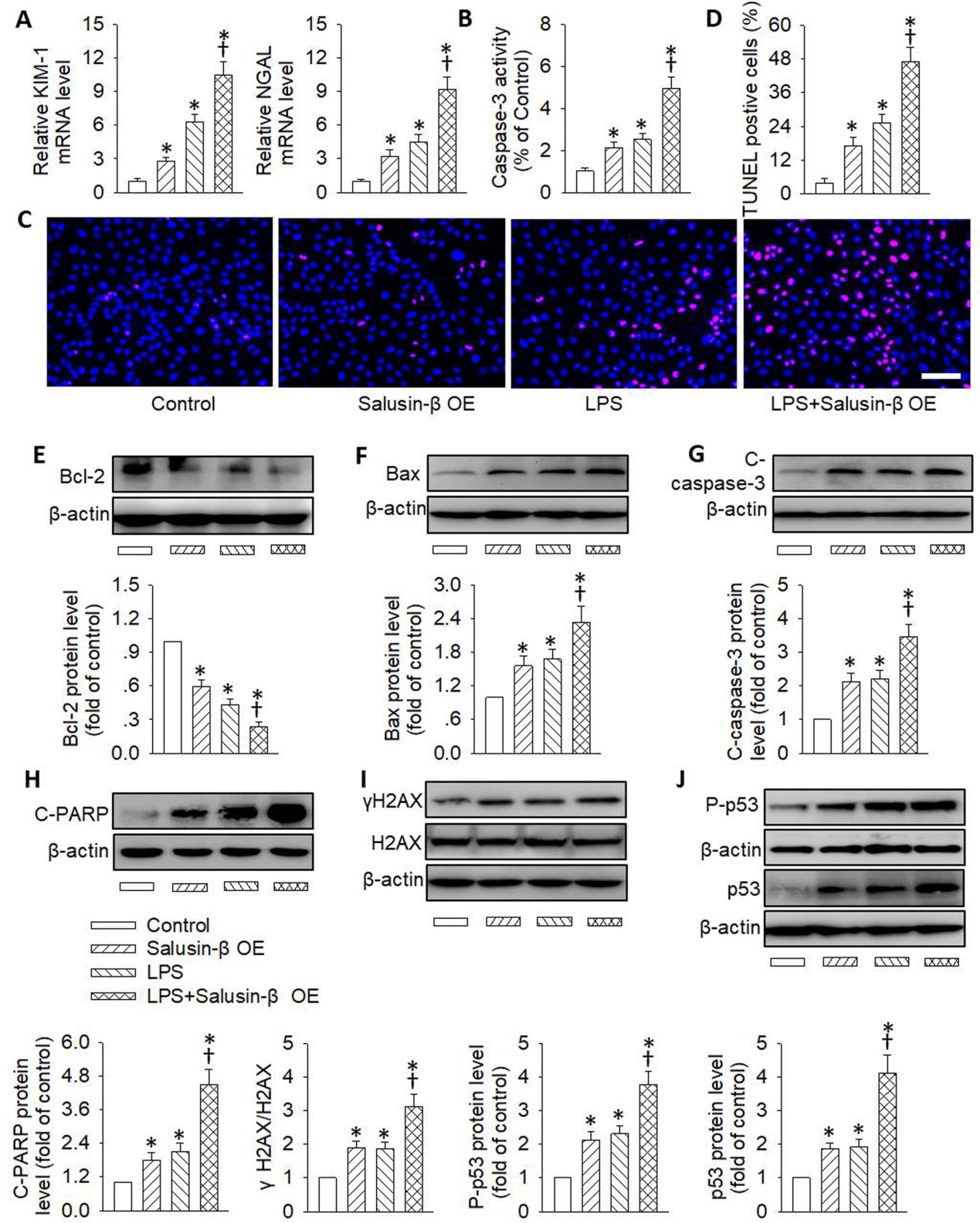


**Fig. S9**. **Effect of salusin-β overexpression on LPS-induced renal tubular cell damage.** HK-2 cells were transfected with lentivirus expressing salusin-β (MOI = 100) for 48 h, and then used for LPS (10 μg/ml) stimulation for 24 h. (**A**) Relative mRNA levels of KIM-1 and NGAL. (**B**) Caspase-3 activity. (**C**) Cell apoptosis determined with TUNEL assay. Blue fluorescence (Hoechst 33342) shows cell nuclei and green fluorescence (TUNEL) stands for apoptotic cells. (**D**) The ratio of TUNEL-positive cells to total cells. (**E**) Representative blots and quantitative analysis of Bcl-2. (**F**) Representative blots and quantitative analysis of Bax. (**G**) Representative blots and quantitative analysis of cleaved-caspase-3 (C-caspase-3). (**H**) Representative blots and quantitative analysis of cleaved-PARP (C-PARP). (**I**) Representative blots and quantitative analysis of γH2AX at 24 h after LPS (10 μg/ml) stimulation. (**J**) Representative blots and quantitative analysis of total and phosphorylated p53. Scale bar=50 μm. Values are mean±SE. * P < 0.05 vs. Control, † P < 0.05 vs. LPS. n = 6 for each group.


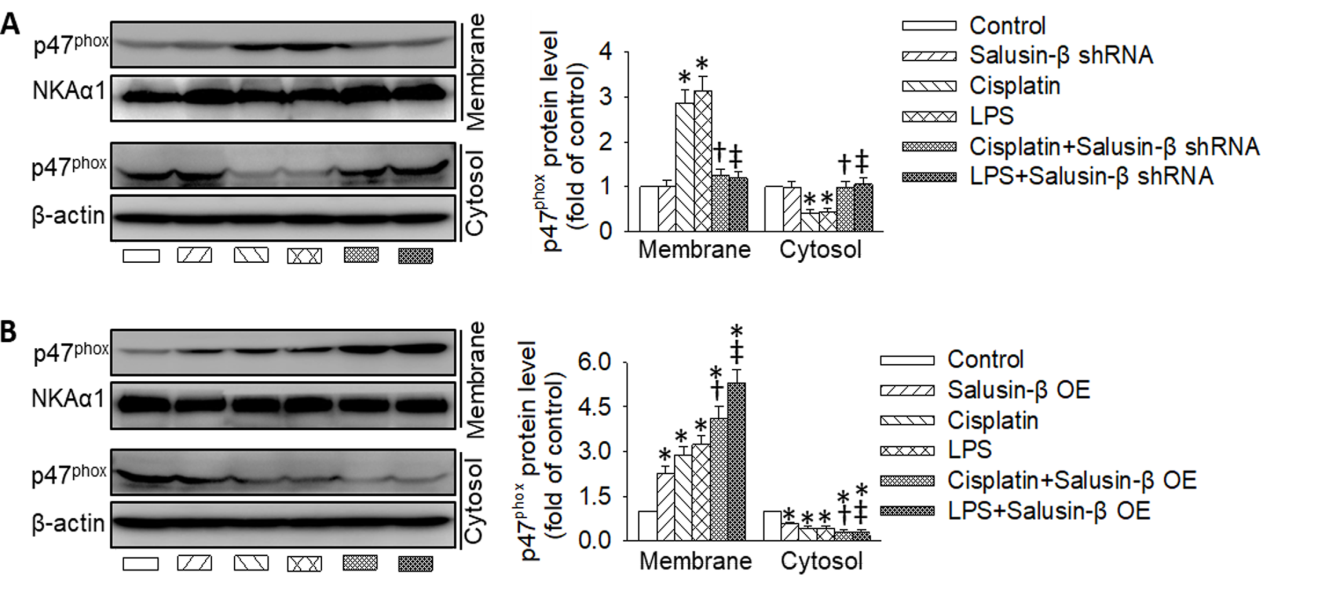


**Fig. S10**. **Effects of salusin-β deficiency/overexpression on the membrane translocation of p47^phox^ in HK-2 cells induced by cisplatin or LPS**. (**A**) The membrane and cytosol level of p47^phox^ in HK-2 cells with salusin-β deficiency in the presence of either cisplatin or LPS. (**B**) The membrane and cytosol level of p47^phox^ in HK-2 cells with salusin-β overexpression in the presence of either cisplatin or LPS. Values are mean±SE. * P < 0.05 vs. Control, † P < 0.05 vs. Cisplatin. ‡ P < 0.05 vs. LPS. n = 6 for each group.


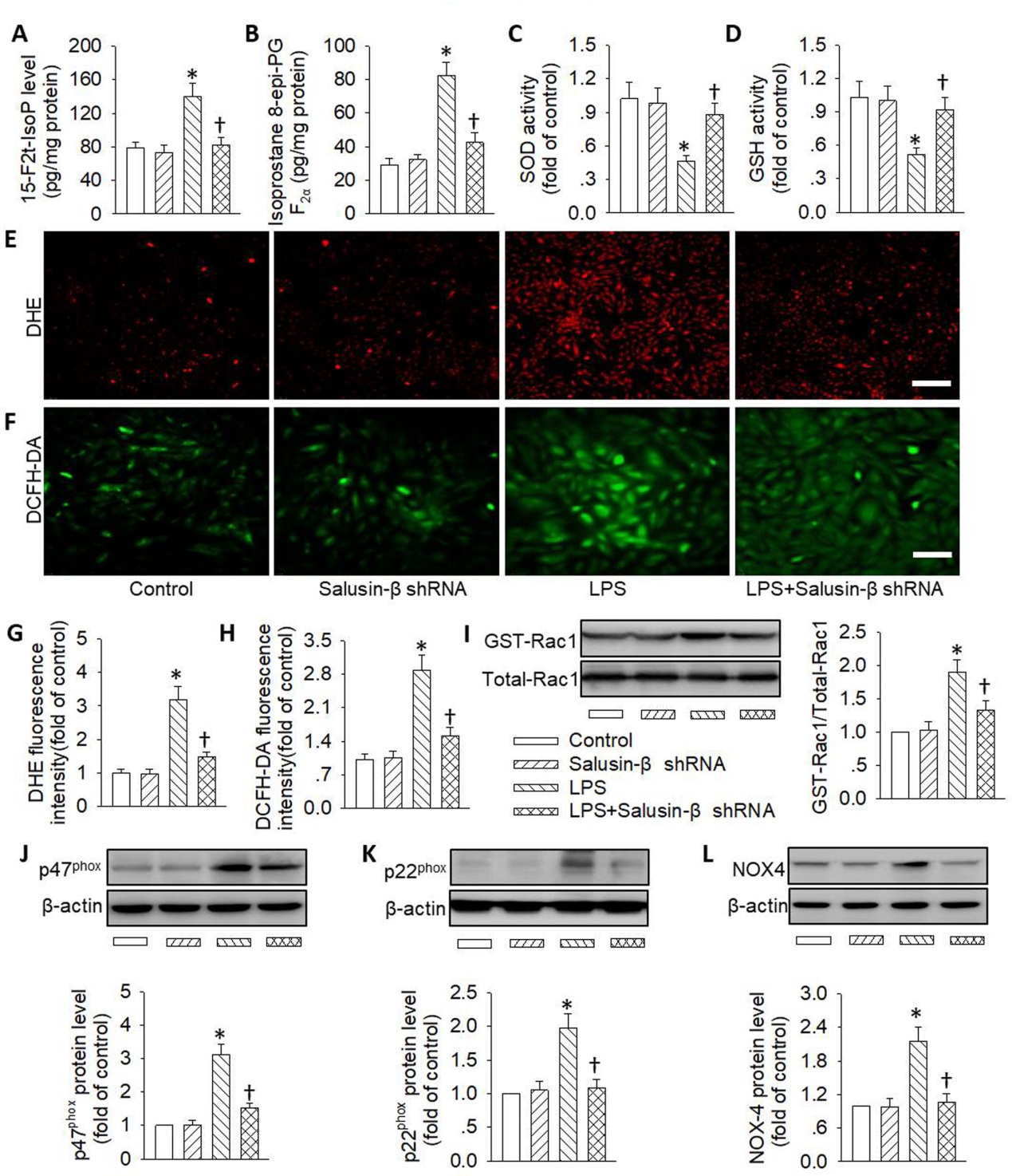


**Fig. S11. Effect of salusin-β knockdown on LPS-induced renal tubular cell oxidative stress.** HK-2 cells were transfected with adenovirus mediated shRNA against salusin-β (MOI = 100) for 48 h, and then used for LPS (10 μg/ml) stimulation for 24 h. (**A**) 15-F2t-isoprostane levels. (**B**) 8-iso-PGF-2α levels. (**C**) SOD activity. (**D**) GSH activity. (**E&G**) Represented images and quantitative analysis showing the levels of superoxide anions detected by DHE staining. (**F&H**) Represented images and quantitative analysis showing the levels of superoxide anions detected by DCFH-DA staining. (**I**) Representative blots and quantitative analysis of GTP-Rac1. (**J**) Representative blots and quantitative analysis of p47^phox^. (**K**) Representative blots and quantitative analysis of p22^phox^. (**L**) Representative blots and quantitative analysis of NOX-4. Scale bar=50 μm. Values are mean±SE. * P < 0.05 vs. Control, † P < 0.05 vs. LPS. n = 6 for each group.


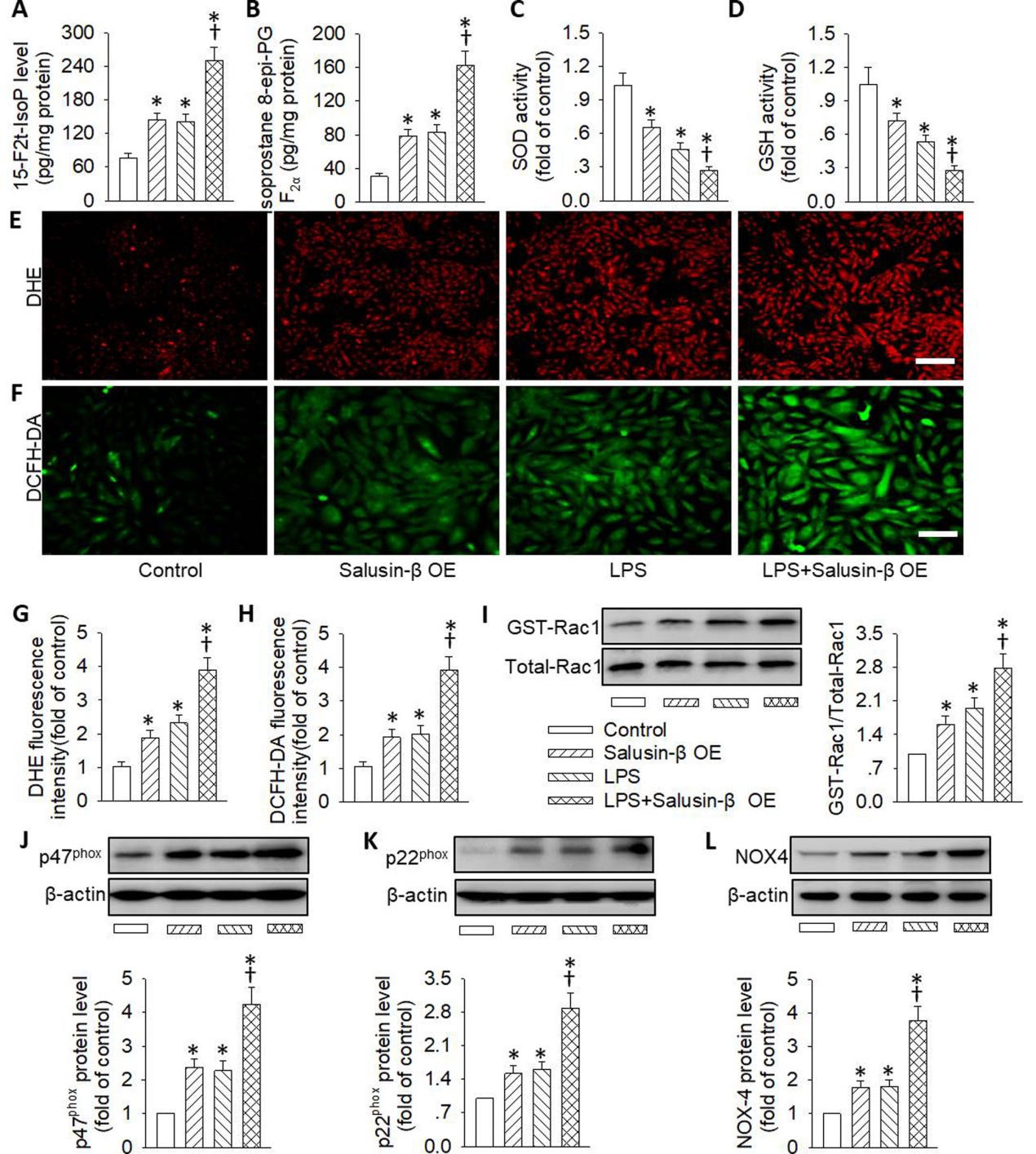


**Fig. S12. Effect of salusin-β overexpression on LPS-induced renal tubular cell oxidative stress.** HK-2 cells were transfected with lentivirus expressing salusin-β (MOI = 100) for 48 h, and then used for LPS (10 μg/ml) stimulation for 24 h. (**A**) 15-F2t-isoprostane levels. (**B**) 8-iso-PGF-2α levels. (**C**) SOD activity. (**D**) GSH activity. (**E&G**) Represented images and quantitative analysis showing the levels of superoxide anions detected by DHE staining. (**F&H**) Represented images and quantitative analysis showing the levels of superoxide anions detected by DCFH-DA staining. (**I**) Representative blots and quantitative analysis of GTP-Rac1. (**J**) Representative blots and quantitative analysis of p47^phox^. (**K**) Representative blots and quantitative analysis of p22^phox^. (**L**) Representative blots and quantitative analysis of NOX-4. Scale bar=50 μm. Values are mean±SE. * P < 0.05 vs. Control, † P < 0.05 vs. LPS. n = 6 for each group.


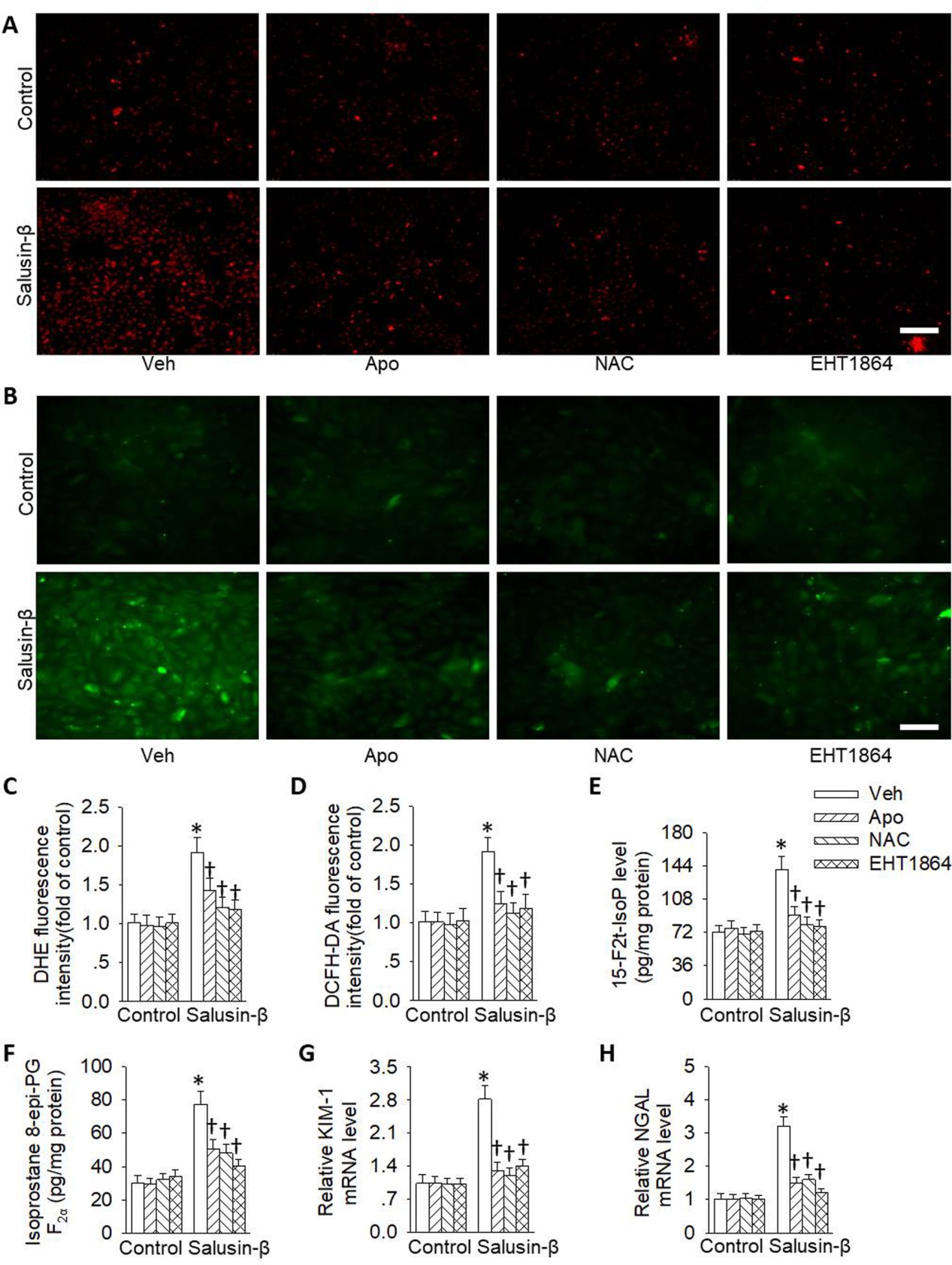


**Fig. S13. Role of the Rac 1/NADPH oxidase/ROS pathway in** **salusin-β-induced tubular cell oxidative injury.** HK-2 cells were pretreated with ROS scavenger NAC (1 mM), NADPH oxidase inhibitor Apo (100 μM), and Rac-1 inhibitor EHT1864 (1 μM) for 30 min, and then transfected with lentivirus expressing salusin-β (MOI = 100) for 48 h. (**A&C**) Represented images and quantitative analysis showing the levels of superoxide anions detected by DHE staining. (**B&D**) Represented images and quantitative analysis showing the levels of superoxide anions detected by DCFH-DA staining. (**E**) 15-F2t-isoprostane levels. (**F**) 8-iso-PGF-2α levels. (**G**) Relative KIM-1 mRNA level. (**H**) Relative NGAL mRNA level. Scale bar=50 μm. Values are mean±SE. * P < 0.05 vs. Control, † P < 0.05 vs. Vehicle (Veh). n = 6 for each group.


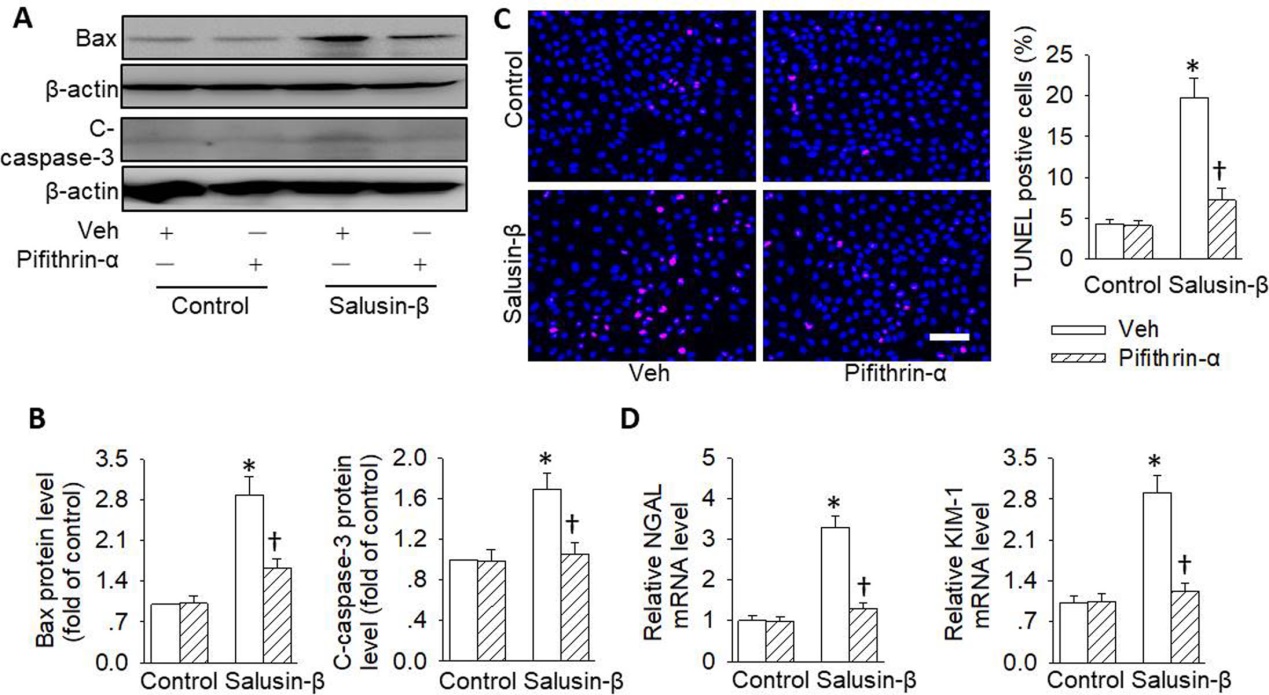


**Fig. S14. Role of p53 activation in** **salusin-β-induced tubular cell injury and apoptosis.** HK-2 cells were pretreated with p53 inhibitor Pifithrin-α (30 μM), and then transfected with lentivirus expressing salusin-β (MOI = 100) for 48 h. (**A**) Representative blots showing the protein level of Bax and cleaved-caspase-3 (C-caspase-3). (**B**) Quantitative analysis of the protein level of Bax and cleaved-caspase-3 (C-caspase-3). (**C**) Cell apoptosis determined with TUNEL assay. Blue fluorescence (Hoechst 33342) shows cell nuclei and green fluorescence (TUNEL) stands for apoptotic cells. (**D**) Relative KIM-1 and NGAL mRNA levels. Scale bar=50 μm. Values are mean±SE. * P < 0.05 vs. Control, † P < 0.05 vs. Vehicle (Veh). n = 6 for each group.


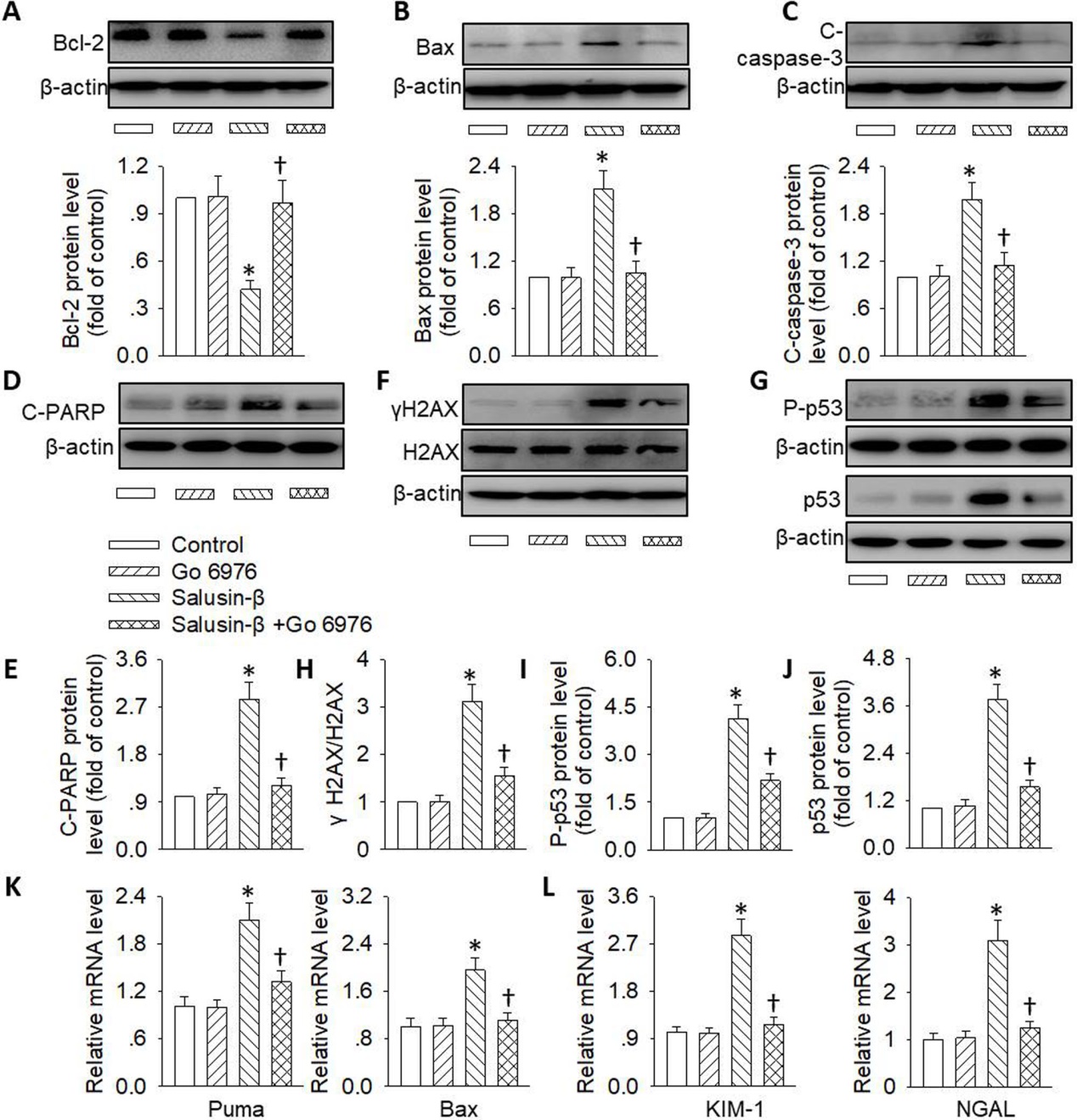


**Fig. S15. Role of the PKC pathway in salusin-β-induced cell apoptosis in renal tubular cells.** HK-2 cells were pretreated with PKC inhibitor Go 6976 (5 μM), and then transfected with lentivirus expressing salusin-β (MOI = 100) for 48 h. The protein expressions of Bcl-2 (**A**), Bax (**B**), cleaved-caspase-3 (C-caspase-3) (**C**), cleaved-PARP (C-PARP) (**D&E**), as well as γH2AX (**F&H**), total and phosphorylated p53 (**G,I&J**) were determined at 48 h after salusin-β overexpression. (**K**) Relative Puma and Bax mRNA levels. (**L**) Relative KIM-1 and NGAL mRNA levels. Values are mean±SE. * P < 0.05 vs. Control, † P < 0.05 vs. Salusin-β. n = 6 for each group.


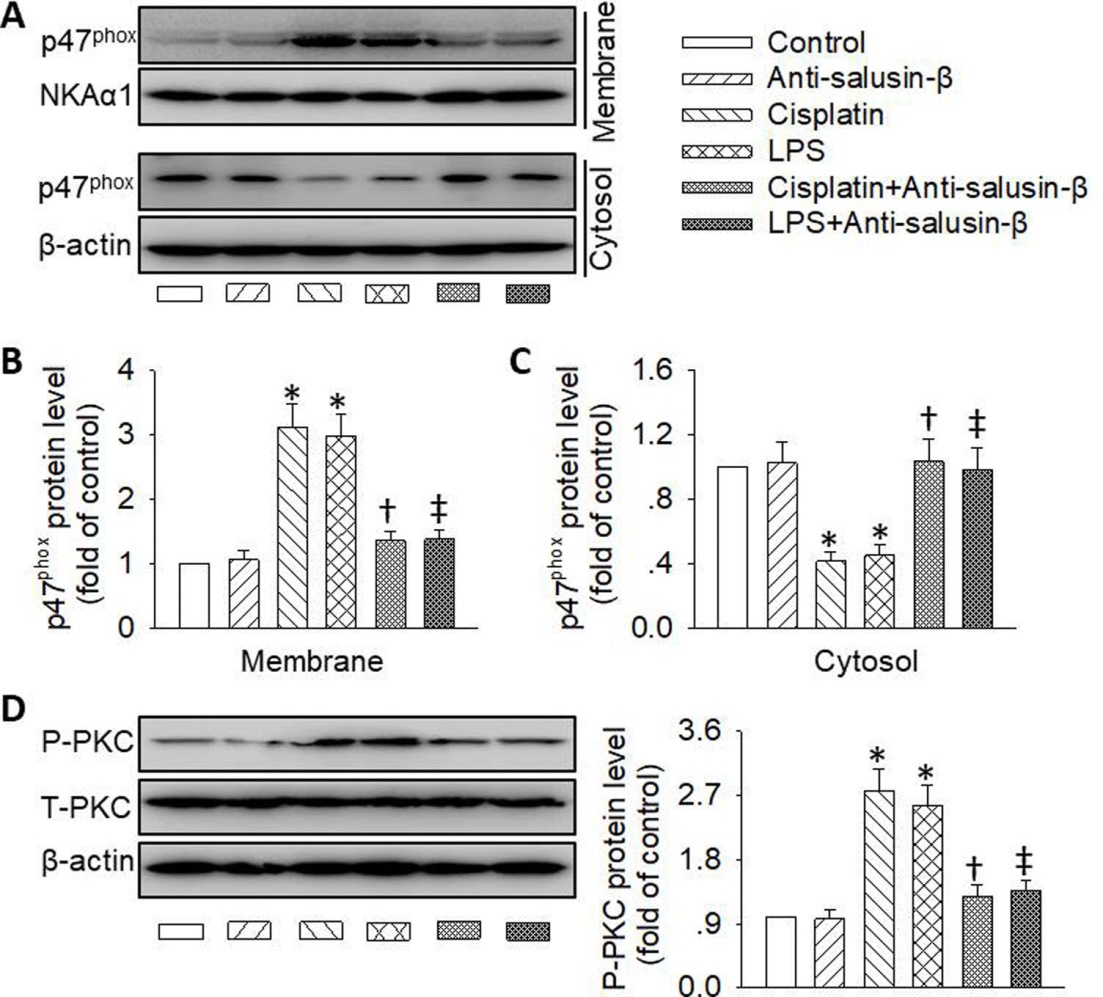


**Fig. S16. Effect of salusin-β inhibition on the membrane translocation of p47phox and phosphorylated PKC in the kidneys after AKI.** (**A-C**) The membrane and cytosol level of p47^phox^. (**D**) The phosphorylated PKC level. Values are mean±SE. * P < 0.05 vs. Control, † P < 0.05 vs. Cisplatin, ‡ P < 0.05 vs. LPS. n = 6 for each group.


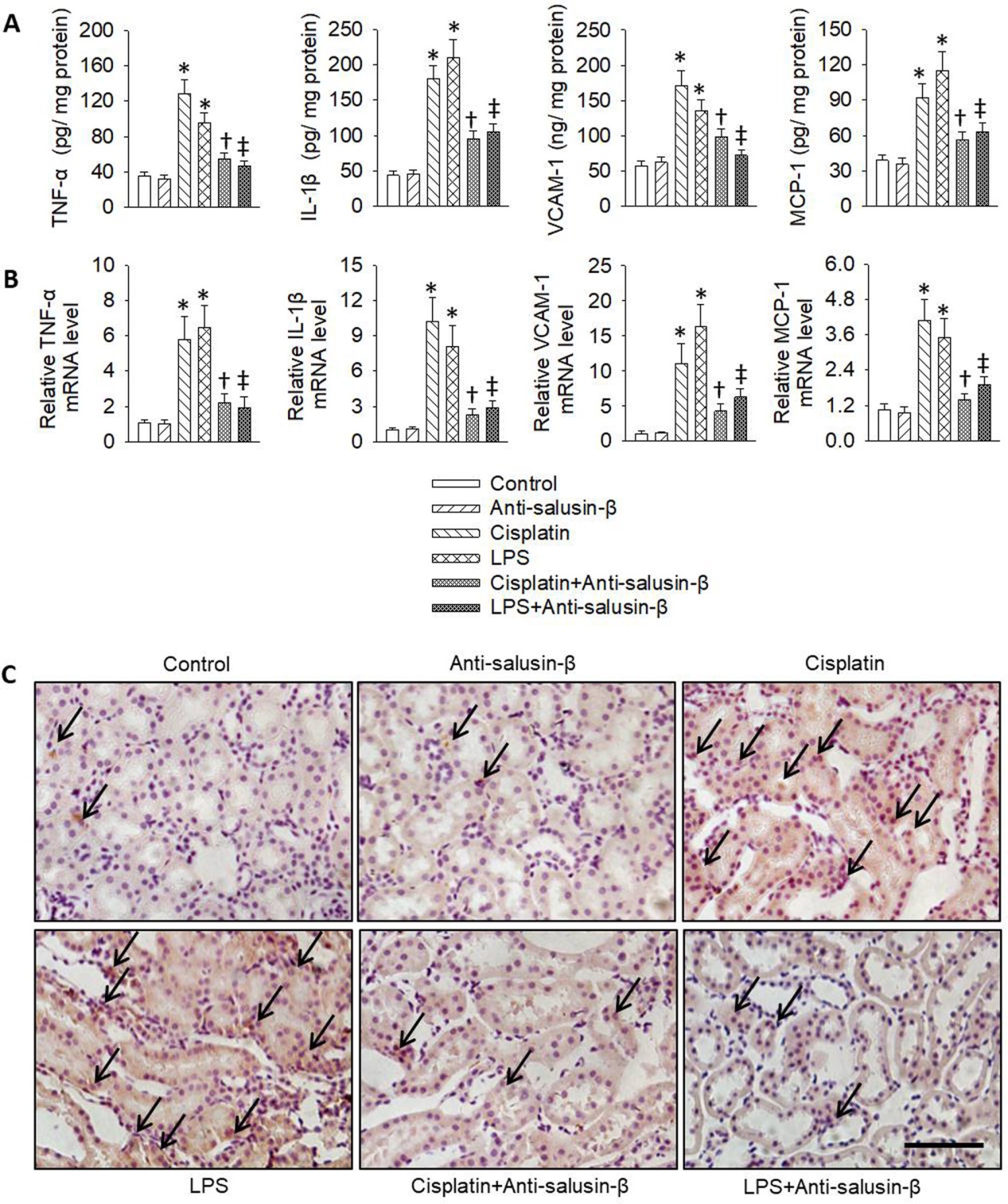


**Fig. S17. Effect of salusin-β inhibition on inflammatory response** **after AKI.** (**A**) Bar group showing the ptotein levels of TNF-α, IL-1β, VCAM-1 and MCP-1 determined with ELISA. (**B**) Bar group showing the mRNA levels of TNF-α, IL-1β, VCAM-1 and MCP-1 determined with real-time PCR. (**C**) Immunohistochemistry showing F4/80-positive macrophages. Scale bar=50 μm. Values are mean±SE. * P < 0.05 vs. Control, † P < 0.05 vs. Cisplatin, ‡ P < 0.05 vs. LPS. n = 6 for each group.

**Table S1. Primer for RT-PCR analysis in HK-2 cells**

| Primers | Sequences (5’-3’) |
| --- | --- |
| GAPDH (Forward) | CCACATCGCTCAGACACCAT |
| GAPDH (Reverse) | CCAGGCGCCCAATACG |
| Salusin-β (Forward) | GGGTGGTATACGGGACCAAT |
| Salusin-β (Reverse) | ACAGCCTGGACAACCTCATC |
| KIM-1 (Forward) | CTGCAGGGAGCAATAAGGAG |
| KIM-1 (Reverse) | ACCCAAAAGAGCAAGAAGCA |
| NGAL (Forward) | TCACCTCCGTCCTGTTTAG |
| NGAL (Reverse) | CTCCTTGGTTCTCCCGTA |
| Bax (Forward) | CATGTTTTCTGACGGCAACTT |
| Bax (Reverse) | CCAGATCACGCCATTTCAC |
| Puma (Forward) | GACCTCAACGCACAGTACGA |
| Puma (Reverse) | GAGATTGTACAGGACCCTCCA |

Note: GAPDH, glyceraldehyde phosphate dehydrogenase; KIM-1: kidney injury molecule 1; NGAL: neutrophil gelatinase-associated lipocalin; Puma: [BCL2 binding component 3 (Bbc3)](https://www.ncbi.nlm.nih.gov/probe/17041707).

**Table S2. Primer for RT-PCR analysis in mice**

| Primers | Sequences (5’-3’) |
| --- | --- |
| GAPDH (Forward) | TCAACGGCACAGTCAAGG |
| GAPDH (Reverse) | ACCAGTGGATGCAGGGAT |
| Salusin-β (Forward) | CACTTCCCCCACCCCAGCCACA |
| Salusin-β (Reverse) | CCGACACTCCGTTCATCTCACT |
| TNF-α (Forward) | GTCCCCAAAGGGATGAGAAG |
| TNF-α (Reverse) | CACTTGGTGGTTTGCTACGA |
| IL-1β (Forward) | CCCAACTGGTACATCAGCACCTC |
| IL-1β (Reverse) | GACACGGATTCCATGGTGAAGTC |
| MCP-1 (Forward) | CCCCACTCACCTGCTGCTACT |
| MCP-1 (Reverse) | TTTACGGGTCAACTTCACATTCAA |
| VCAM-1 (Forward) | GCCCTCACTTGCAGCACTAC |
| VCAM-1 (Reverse) | TCCTCACCTTCGCGTTTAGT |
| KIM-1 (Forward) | ACATATCGTGGAATCACAACGAC |
| KIM-1 (Reverse) | ACTGCTCTTCTGATAGGTGACA |
| NGAL (Forward) | GCAGGTGGTACGTTGTGGG |
| NGAL (Reverse) | CTCTTGTAGCTCATAGATGGTGC |
| Bax (Forward) | TGGAGATGAACTGGACAGCAATAT |
| Bax (Reverse) | GCAAAGTAGAAGAGGGCAACCAC |
| Puma (Forward) | CGGCGGAGACAAGAAGA |
| Puma (Reverse) | CACCTAGTTGGGCTCCATTT |

Note: GAPDH, glyceraldehyde phosphate dehydrogenase; TNF-α, tumor necrosis factor-α; IL-1β, interleukin-1β; MCP-1, monocyte chemoattractant protein 1; VCAM-1, vascular cellular adhesion molecule-1; KIM-1: kidney injury molecule 1; NGAL: neutrophil gelatinase-associated lipocalin; Puma: [BCL2 binding component 3 (Bbc3)](https://www.ncbi.nlm.nih.gov/probe/17041707).
